# Inhibition of BRD4 Attenuates ER Stress-induced Renal Ischemic-Reperfusion Injury

**DOI:** 10.7150/ijbs.83040

**Published:** 2024-02-11

**Authors:** Paula Diaz-Bulnes, Ramon M Rodríguez, Elisenda Banon-Maneus, María Laura Saiz, Cristian Ruiz Bernet, Viviana Corte-Iglesias, Maria Jose Ramirez-Bajo, Marta Lazo-Rodriguez, Isaac Tamargo-Gómez, Raúl R. Rodrigues-Diez, Ana B Sanz, Carmen Diaz-Corte, Marta Ruiz-Ortega, Fritz Diekmann, Ana M Aransay, Carlos Lopez-Larrea, Beatriz Suarez-Alvarez

**Affiliations:** 1 Translational Immunology, Instituto de Investigación Sanitaria del Principado de Asturias (ISPA), 33011 Oviedo, Spain.; 2 RICORS2040 (Kidney Disease), Instituto de Salud Carlos III, 28029 Madrid, Spain.; 3 Lipids in Human Pathology, Health Research Institute of the Balearic Islands (IdISBa), Research Unit, University Hospital Son Espases, 07120 Palma, Spain.; 4 Laboratori Experimental de Nefrologia i Trasplantament (LENIT), Institut d'Investigacions Biomèdiques August Pi i Sunyer (IDIBAPS), Barcelona, Spain.; 5 Autophagy and Metabolism Laboratory, Instituto de Investigación Sanitaria del Principado de Asturias (ISPA), Oviedo, Spain.; 6 Laboratorio de Nefrología Experimental, Instituto de Investigación Sanitaria-Fundación Jiménez Díaz, Universidad Autónoma de Madrid, Madrid, Spain.; 7 Servicio de Nefrología, Hospital Universitario Central de Asturias, Oviedo, Asturias, Spain.; 8 Cellular Biology in Renal Diseases Laboratory, IIS-Fundación Jiménez Díaz, Universidad Autónoma Madrid, 28040 Madrid, Spain.; 9 Departmento de Nefrologia y Transplante Renal, Institut Clínic de Nefrologia i Urologia, Hospital Clínic Barcelona, Barcelona, Spain; 10 Genome Analysis Platform, CIC bioGUNE-BRTA, Derio, Spain.; 11 Centro de Investigación Biomédica en Red en el Área Temática de Enfermedades Hepáticas (CIBERehd), Madrid, Spain.

**Keywords:** BRD4, ER stress, UPR, renal damage, Hypoxia

## Abstract

Renal ischemia-reperfusion injury (IRI) leads to endoplasmic reticulum (ER) stress, thereby initiating the unfolded protein response (UPR). When sustained, this response may trigger the inflammation and tubular cell death that acts to aggravate the damage. Here, we show that knockdown of the BET epigenetic reader BRD4 reduces the expression of ATF4 and XBP1 transcription factors under ER stress activation. BRD4 is recruited to the promoter of these highly acetylated genes, initiating gene transcription. Administration of the BET protein inhibitor, JQ1, one hour after renal damage induced by bilateral IRI, reveals reduced expression of *ATF4* and *XBP1* genes, low KIM-1 and NGAL levels and recovery of the serum creatinine and blood urea nitrogen levels. To determine the molecular pathways regulated by ATF4 and XBP1, we performed stable knockout of both transcription factors using CRISPR-Cas9 and RNA sequencing. The pathways triggered under ER stress were mainly XBP1-dependent, associated with an adaptive UPR, and partially regulated by JQ1. Meanwhile, treatment with JQ1 downmodulated most of the pathways regulated by ATF4 and related to the pathological processes during exacerbated UPR activation. Thus, BRD4 inhibition could be useful for curbing the maladaptive UPR activation mechanisms, thereby ameliorating the progression of renal disease.

## Introduction

Tubular epithelial cells (TECs) are especially vulnerable to a variety of insults, such as hypoxia, drugs, toxins and proteinuria. They respond to injury not only by triggering mechanisms and pathways to restore cellular homeostasis, but also by inducing the production of inflammatory and pro-fibrotic factors that determine the progression of chronic kidney disease (CKD) 1. Protein homeostasis is disturbed by renal ischemia-reperfusion injury (IRI), inducing the accumulation of unfolded proteins within the endoplasmic reticulum (ER) of tubular cells 2. When proteostasis is disrupted and unfolded proteins accumulate, ER stress initiates the unfolded protein response (UPR) that attenuates protein synthesis, eliminates misfolded proteins and enhances the ability of the ER to fold proteins, allowing cell survival 3. However, under sustained ER stress, the adaptive ability of UPR is lost, leading to cell death by apoptosis. Targeting prolonged UPR activation might offer protection from renal damage and its progression 4,5. Pharmacological inhibition of ER stress with the chemical chaperones TUDCA (tauroursodeoxycholic acid) and 4-PBA (4-phenylbutyric acid) reduces inflammation and tubular cell death, alleviating damage and preventing renal failure 6, 7.

Under ER stress, the resident chaperone BiP/GRP78 (78-kDa glucose-related protein) engages with unfolded proteins to release three major sensors: IRE1α, PERK and ATF6. IRE1α allows processing of XBP1 to a splicing form (XBP1s) that is functionally active as a transcription factor and that induces JNK, NF-κB and NLRP3-inflammasome activation 8-10. The PERK branch phosphorylates and activates eukaryotic initiation factor 2 alpha (eIF2α), enabling the expression of ATF4. This transcription factor helps regulate autophagy and promote the expression of the pro-apoptotic factor CHOP (C/EBP-homologous protein), and is a metabolic effector of mTORC1 11, 12. The third sensor, ATF6, is transported to the Golgi, where it is cleaved and moves to the nucleus, where it induces expression of XBP1 and CHOP, among others 13, 14. Therefore, each branch of the UPR pathway facilitates the transcription of many genes, some of which restore ER homeostasis, while others aggravate the damage.

BRD4, the best characterized member of the bromodomain and extra-terminal (BET) protein family, recognizes and binds to acetylated lysine on histones and other non-histone proteins and recruits transcriptional complexes to gene promoters to initiate this transcription 15. Therefore, pharmacological targeting of BRD4 has been proposed as an alternative therapy for treating a wide range of human diseases, including those of the kidney 16, 17. We and other groups have reported that BRD4 regulates the expression of pro-inflammatory genes, blocks the NF-κB pathway and NLRP3-inflammasome activation, and modulates the neutrophil activation associated with renal injury 18-20. Blockage of BRD4 has also been shown to be a potent antifibrotic therapy in renal cells and in a mouse model of UUO-induced renal damage 21, 22. However, its role in modulating UPR genes and the consequent pathological processes associated with renal damage has not yet been explored.

Here, we set out to determine the role of BRD4 in regulating UPR genes and the underlying mechanisms generated as a consequence of renal ischemic damage. We explore the outcome of BRD4 targeting using the JQ1 inhibitor in an *in vivo* IRI model, and show that BRD4 is a potential target for downregulating ATF4 and XBP1 expression, and for alleviating the ischemic renal damage produced by sustained ER stress.

## Methods

### Cell culture and treatments

Human proximal tubular epithelial cells (TECs) (HK-2 cell line; CRL-2190; ATCC, VA) were maintained in RPMI 1640 medium (Gibco, Carlsbad, CA, USA) supplemented with 10% fetal bovine serum, 1% penicillin/streptomycin, 1% insulin transferrin selenite (Gibco) and 5 ng/ml hydrocortisone (Sigma-Aldrich, St. Louis, MO, USA). Cells were cultured at 0.25x10^6^ cells/ml in serum-free medium for 24 h before the assay and further stimulated with thapsigargin (Tg; 4 μM; 24 h; Sigma-Aldrich). Inhibitors used were: JQ1(+) or its inactive enantiomer JQ1(-) (100-500 nM; 24 h; Selleckchem, Houston, TX, USA); I-BET 726 (400-1000 nM; 24 h; Cayman Chemical, Ann Arbor, MI, USA) and 5,6-dichlorobenzimidazole 1-β-D-ribofuranoside DRB (20-60 μM; 6 h; Sigma-Aldrich). For hypoxic conditions, cells were cultured in medium without nutrients for 12 h in a hypoxic incubator (1% O_2_, 94% N_2_ and 5% CO_2_, 37°C). In some conditions, HK-2 cells were followed by reoxygenation at different times (2, 4, and 6 h) with fresh complete medium, and in 5% CO_2_ and 95% air at 37°C.

### IR injury animal model and animal studies

All animal procedures were performed in accordance with the guidelines for animal research in the European Community and with the approval of the local government authorities at IDIBAPS, Hospital Clinic, Barcelona. Briefly, bilateral ischemia reperfusion injury (IRI) was induced in 9-12-week-old male C57BL/6 mice (Charles River Laboratories, Wilmington, MA, USA) by clamping both renal pedicles for 45 min at 37°C, adopting a retroperitoneal approach, under isofluorane anesthesia. After removing the clamps, reperfusion was verified by visual inspection of the kidney. Following surgery, mice were randomly assigned to three groups (n=5/group): Sham, IRI and IRI + JQ1. Sham group mice underwent the same procedure except the renal pedicles were not clamped. The BET bromodomain inhibitor, JQ1(+), supplied by James Bradner's laboratory (Dana-Farber Cancer Institute, Boston, MA, USA), was dissolved in 10% hydroxypropyl β-cyclodextrin (Sigma Aldrich) and administered by intraperitoneal injection of a single dose of 100 mg/kg 1 h after surgery. Mice were euthanized at different times after reperfusion, and the kidneys and blood collected for further analysis.

### Assessment of renal injury

Kidney sections (2-5 μm) were stained with periodic acid-Schiff (PAS) reagent to assess renal injury and quantify the percentage of necrotic tubules by an experienced pathologist and an observer blinded to the experimental conditions. Images were acquired with a Leica DFC700T optical microscope (Leica Biosystems, Wetzlar, Germany). Blood samples were collected in heparin tubes and serum was obtained from whole blood after centrifugation. Serum creatinine and urea were determined using a creatinine assay kit (Abcam, Cambridge, UK) and a blood urea nitrogen (BUN) colorimetric detection kit (Arbor Assays, Ann Arbor, MI, USA), respectively, and according to the manufacturer's instructions.

### Gene expression studies

Total RNA from HK-2 cells or frozen kidneys was isolated using a GeneMATRIX Universal RNA purification kit (EURx, Gdansk, Poland) following the manufacturer's instructions. Purified RNA (1 µg) was reversed-transcribed to cDNA using a high-capacity cDNA reverse-transcription kit (Applied Biosystems, Foster City, CA, USA). Quantification was performed by the reverse-transcription polymerase chain reaction (RT-PCR) using TB Green Premix Ex TaqII (Takara Bio Inc., Kusatsu, Japan) or TaqMan Gene Expression Master Mix (Applied Biosystems), and analyzed with a StepOnePlus^TM^ Real-Time PCR System (Applied Biosystems). Expression of each gene of interest was normalized to the housekeeping gene *Gapdh* and data were calculated by the ΔCt method. The primers and TaqMan assays used are listed in **Table S1.**

### CRISPR-Cas9

HK-2 cells were transfected with a single guide RNA (sgRNA) specific to ATF4 and XBP1 and designed using the https://cctop.cos.uni-heidelberg.de website. Plasmids expressing Cas9 and sgRNA were generated by inserting custom sgRNA into lentiCRISPRv2 (#52961; Addgene, Watertown, MA, USA) following the manufacturer's instructions. Briefly, lentiviruses were packaged in HEK 293T cells by transfection with specific sgRNA/Cas9-expressing lentiCRISPRv2 and the packaging plasmid psPAX-2 (#12260; Addgene) and psPMD2g (#12259; Addgene) at 1:1:1 molar ratio using the Lipofectamine 3000 Reagent (Invitrogen). Medium was replaced after 6 h and virus samples collected at 24, 48 and 72 h. HK-2 cells were infected with lentivirus in the presence of polybrene (8 μg/ml) and further selected with 1 μg/ml of puromycin to obtain single clones. sgRNAs and primers are listed in **Table S2.**

### Gene silencing

HK-2 cells were transfected with variable quantities of ON-TARGETplus siRNAs specific to human BRD4 (L-004937-00-0005) or of the non-targeting control pool as a negative control (D-001810-10-05) using DharmaFECT1 Transfection Reagent. All reagents were supplied by GE Healthcare Dharmacon, Lafayette, CO, USA.

### Protein analysis

Proteins were extracted using radioimmunoprecipitation assay (RIPA) lysis buffer supplemented with a protease and phosphatase inhibitor cocktail (Merck Millipore, Burlington, MA, USA) for 30 min on ice, quantified using the Bradford protein assay (Bio-Rad), and separated on 10-12% polyacrylamide-SDS gels. Membranes were incubated overnight at 4°C with the following primary antibodies: XBP1 spliced form (NBP2-20917, 1:1000; Novus Biologicals, Centennial, CO, USA), ATF4 (11815S, 1:1000; Cell Signaling Technology, Danvers, MA, USA), ATF6 (MAB71527-SP, 1:2000; R&D Systems, Minneapolis, MI, USA), BRD4 (A301-985A; Bethyl Laboratories, Montgomery, TX, USA), KIM-1 (AF1817, 1:1000; R&D Systems), NGAL (Sc-50350, 1:500; Santa Cruz Biotechnology, Santa Cruz, CA, USA), and β-actin (4967S, 1:2000; Cell Signaling Technology); followed by incubation with HRP-conjugated IgG secondary antibody for 1 h. Immunostained bands were visualized using a chemiluminescence kit (Luminata Forte Western HRP Substrate; Merck Millipore), detected on an ImageLab system (Bio-Rad, Hercules, CA, USA), and quantified with ImageJ version 1.53c software (NIH Image).

### Chromatin immunoprecipitation assay

HK-2 cells (10^7^) were fixed with 1% formaldehyde (Sigma-Aldrich) in RPMI medium for 30 min at 4°C, followed by quenching with 0.125 M glycine for 10 min at 4°C. Further, chromatin was shared on a BioRuptor (Diagenode, Liège, Belgium) into DNA fragments between 500-1000 bp long. 100 μg of chromatin was diluted into ChIP dilution buffer (0.01% SDS; 1.1% Triton X100; 1.2 mM EDTA; 16.7 mM Tris-HCL, pH 8.1 and 167 mM NaCl) containing protease and phosphatase inhibitor (Thermo Scientific) and incubated with specific antibodies to BRD4 (A301-985A; Bethyl Laboratories), anti-phospho RNA pol II (Ser 2) (MABE953; Merck), acetylated H3 (06-599; Merck), acetylated H4 (06-598; Merck) or normal rabbit IgG (A300-109A; Bethyl Laboratories) overnight at 4°C. Antibody-chromatin complexes were recovered with Salmon Sperm DNA/Protein A-Agarose beads (Merck) for 1 h at 4°C, washed and eluted from the beads with elution buffer (1% SDS, 0.1 M NaHCO_3_). After reverse-crosslinking and proteinase K treatment, DNA was extracted and analyzed by quantitative RT-PCR using specific primers (**Table S1**). Chromatin obtained before immunoprecipitation was used as the input control. For ChIP assay in kidney samples, kidneys were immediately fixed with 1% paraformaldehyde after extraction, quenched with 0.125 M glycine and stored at -80°C until use. The High Cell ChIP Kit (Diagenode) was used in accordance with the manufacturer's instructions, employing specific primers (**Table S1**). In both assays, enrichment was calculated using the formula: ΔCt=Ct (bound) - [Ct (input) - log_2_ (Input dilution factor)] and data are expressed as the fold enrichment (FE) of each specific antibody relative to the negative control, where FE=2 exp - [ΔCt (specific antibody) - ΔCt (normal IgG)].

### RNA sequencing and gene expression analysis

RNA from HK-2 cells was isolated with a PureLink™ RNA Mini Kit (Invitrogen, Carlsbad, CA, USA). The integrity of the RNA was analyzed in RNA 6000 Nano Chips with a Bioanalyzer 2100 (Agilent Technologies, Santa Clara, CA, USA) and quantified using a fluorometric Qubit RNA HS assay kit (Invitrogen, Carlsbad, CA, USA). Briefly, starting from 1 µg of total RNA, sequencing libraries were prepared following the TruSeq Stranded mRNA Sample Preparation Guide (Part #15031058 Rev. E), using the TruSeq Stranded mRNA Library Prep kit (Illumina Inc., Cat. #20020294) and TruSeq RNA Single Indexes (Illumina Inc., Cat. #20020492 and 20020493). Sequencing was carried out using a HiSeq 2500 platform (Illumina Inc.). Files of data generated by the sequencing were aligned against the human genome (Homo_sapiens.GRCh38.94.gtf) using the STAR program 23, and the genes and transcripts were analyzed with the RSEM program 24 using GENCODE v26 25. Expression data were analyzed using R package, the DEQseq2 method of the Bioconductor project (www.bioconductor.org), and lumi packages. To detect genes differentially expressed between groups, the moderated t-test was used. Genes with a >2-fold change and an adjusted p<0.05 were considered significant. Functional interaction network data were obtained from STRING v10 26, generated using the Fruchterman-Reingold clustering algorithm, and then built using Cytoscape software 27. The resulting network was exported to Gephi (http://gephi.org). Gene functional analysis was carried out using the DAVID Go web-based tool 28. Raw mRNAseq data have been deposited in the NCBI Gene Expression Omnibus under accession number GEO:GSE217103.

### Statistical analysis

Data were summarized as the mean ± standard error of the mean (SEM) of at least two or three independent experiments. Characteristics of groups were compared using Fisher´s exact test for categorical variables, and the Wilcoxon paired-samples test, or the Mann-Whitney U test for unpaired samples. Statistical analyses were carried out with IBM SPSS Statistics, version 27.0 (IBM SPSS, Armonk, NY, USA) and GraphPad-Prism v7 (San Diego, CA, USA). Statistical significance was concluded for values of p<0.05. Statistical details of the experiments and significance are noted in the respective figures and legends.

## Results

### Blockage of BET proteins attenuates UPR activation triggered in human TECs

**Figure 1:**
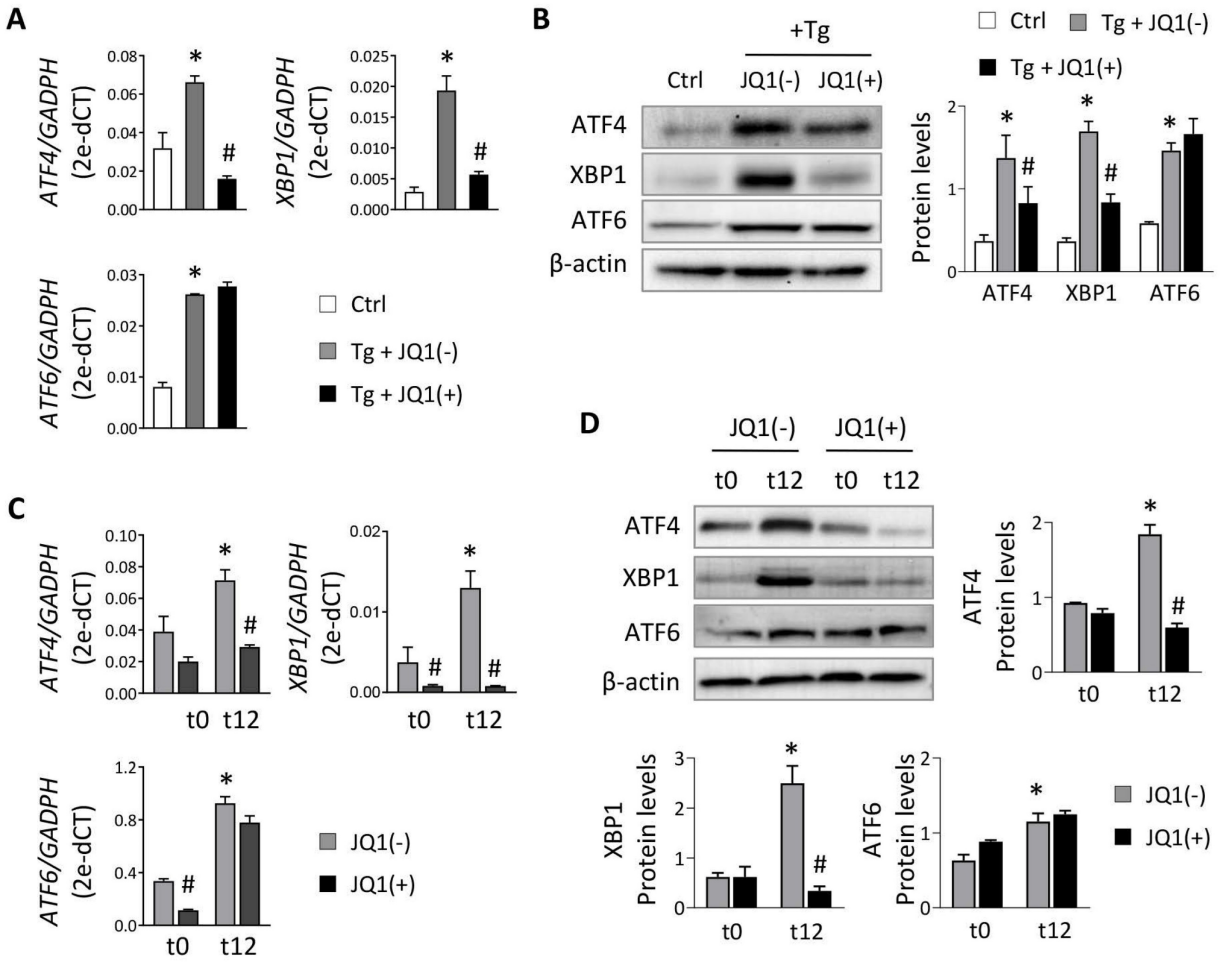
** Treatment with JQ1 ameliorates overexpression of UPR genes induced by thapsigargin and hypoxia in HK-2 cells.** Renal TECs (HK-2 cell line) were treated with DMSO (Ctrl) or Tg (4 μM, 24 h) (**A,B**) or cultured under hypoxic conditions (1% O_2_, 5% CO_2_) at different times (t0, normoxia; t12, 12 h) (**C,D**). The inhibitor of BET proteins, JQ1(+), or its inactive enantiomer, JQ1(-), were co-cultured at the same indicated times under both cultured conditions. Gene expression levels of *ATF4*, *XBP1* and *ATF6* were analyzed by RT-PCR (**A,C**) and protein levels were analyzed by western blot (**B,D**). Data are expressed as the mean ± SEM of at least three independent experiments. *GADPH* and β-actin were used as housekeeping markers of RT-PCR and western blot, respectively. Statistical analyses involved use of the two-tailed Student's paired t-test and the Wilcoxon test. *p<0.05 *vs*. control (DMSO) or t0 (normoxia) and # *vs.* cells treated with JQ1(-) + Tg or cells in hypoxia (t12) treated with JQ1(-).

To evaluate the role of BET proteins in UPR pathway activation in tubular cells, we used HK-2 cells treated with thapsigargin (Tg, 4 μM), an inhibitor of the sarco/endoplasmic reticulum Ca^2+^ ATPase (SERCA) that impairs calcium access to the ER, inducing the three branches of the UPR pathway. After Tg stimulation, increased expression of the three downstream transcription factors (ATF4, XBP1 and ATF6) was detected (**Figure 1A**). For XBP1 only the active XBP1s form was analyzed (and referenced as XBP1 from now on). The induction of these genes declined significantly and dose-dependently when JQ1(+) was added (**Figure 1A and Figure S1A**), except for ATF6, whose expression levels remained unchanged. Similar results were observed at the protein level (**Figure 1B**) and with other BET protein inhibitors, such as I-BET762 (**Figure S1B**). Additionally, specific BRD4 silencing significantly decreased the induced ATF4 and XBP1 expression, but not ATF6, after sustained UPR activation with Tg (**Figure S1C and S1D**).

**Figure 2:**
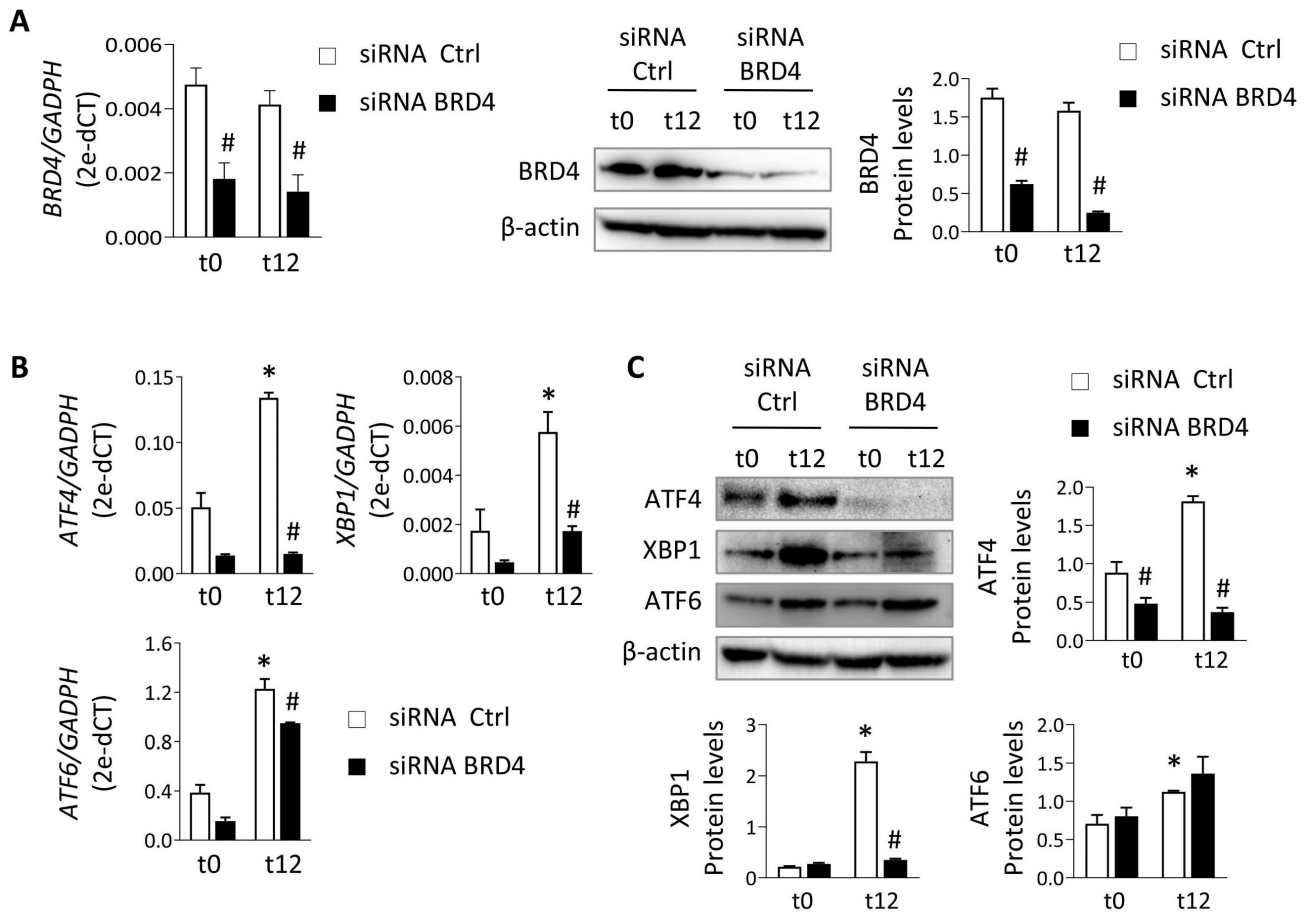
** Specific BRD4 silencing impairs expression of UPR genes induced under hypoxia.** HK-2 cells were transfected with a specific siRNA against BRD4 (siRNA BRD4) or control siRNA (siRNA Ctrl, 40 nM, 48 h) before exposure to conditions of normoxia (t0) or hypoxia (t12). (**A**) Transcriptional and protein levels of BDR4 after specific silencing. Transcriptional levels of *ATF4*, *XBP*1 and *ATF6* were determined by RT-PCR (**B**) and protein levels (**C**) assayed by western blot. Data are expressed as mean ± SEM of three independent experiments. Statistical analyses involved use of the two-tailed Student's paired t-test and the Wilcoxon test. *p<0.05 *vs.* t0 (normoxia), # *vs.* siRNA Ctrl-treated cells.

To verify these data under physiological conditions, HK-2 cells were subjected to different periods of hypoxia and the highest expression of ATF4, XBP1 and ATF6 was detected at 12 h (t12) (**Figure S2**). At this time, when cells were treated with the JQ1(+) inhibitor, the expression of ATF4 and XBP1 was not induced, suggesting that their regulation was dependent on BRD4 (**Figure 1C and 1D**). These results were corroborated using specific siRNA silencing of BRD4 (**Figure 2A**), where the increased ATF4 and XBP1 expression observed under hypoxic conditions (t12) was again reduced after BRD4 blockage reaching similar levels to that detected in normoxia (t0) (**Figure 2B and 2C**). Similarly to what was observed when induced with Tg, the ATF6 protein levels remained unchanged (**Figure 2C**).

The cellular response to hypoxia and reoxygenation (H/R) is a complex and tightly regulated process. We aimed to determine whether the downregulation of ATF4 and XBP1 by JQ1(+) is maintained after reperfusion. Our findings demonstrate an increase of the transcription levels of ATF4 and XBP1 after 2 h of reoxygenation, that are subsequently sustained (**Figure S3A**). These results were confirmed at the protein level, although ATF4 reverts to hypoxic levels 6 h after reperfusion (**Figure S3B**). Once again, the treatment with JQ1(+) effectively prevents the upregulation of both ATF4 and XBP1 during reoxygenation (**Figure S3A and S3B**).

In order to evaluate whether the activation of the UPR pathway under these conditions induces changes in BRD4 expression, we evaluated the transcriptional and protein levels of BRD4 expression in HK-2 cells treated with Tg (**Figure S4A**) or cultured under hypoxic and H/R conditions. (**Figure S4B and S4C**). Across all conditions, BRD4 levels remained unaltered compared to the basal conditions, suggesting that its role in the cellular response to ER stress may not be primarily governed by changes in its abundance but rather by its binding to specific regulatory regions of these genes.

### BRD4 is recruited to promoter regions of UPR genes to induce their expression

**Figure 3:**
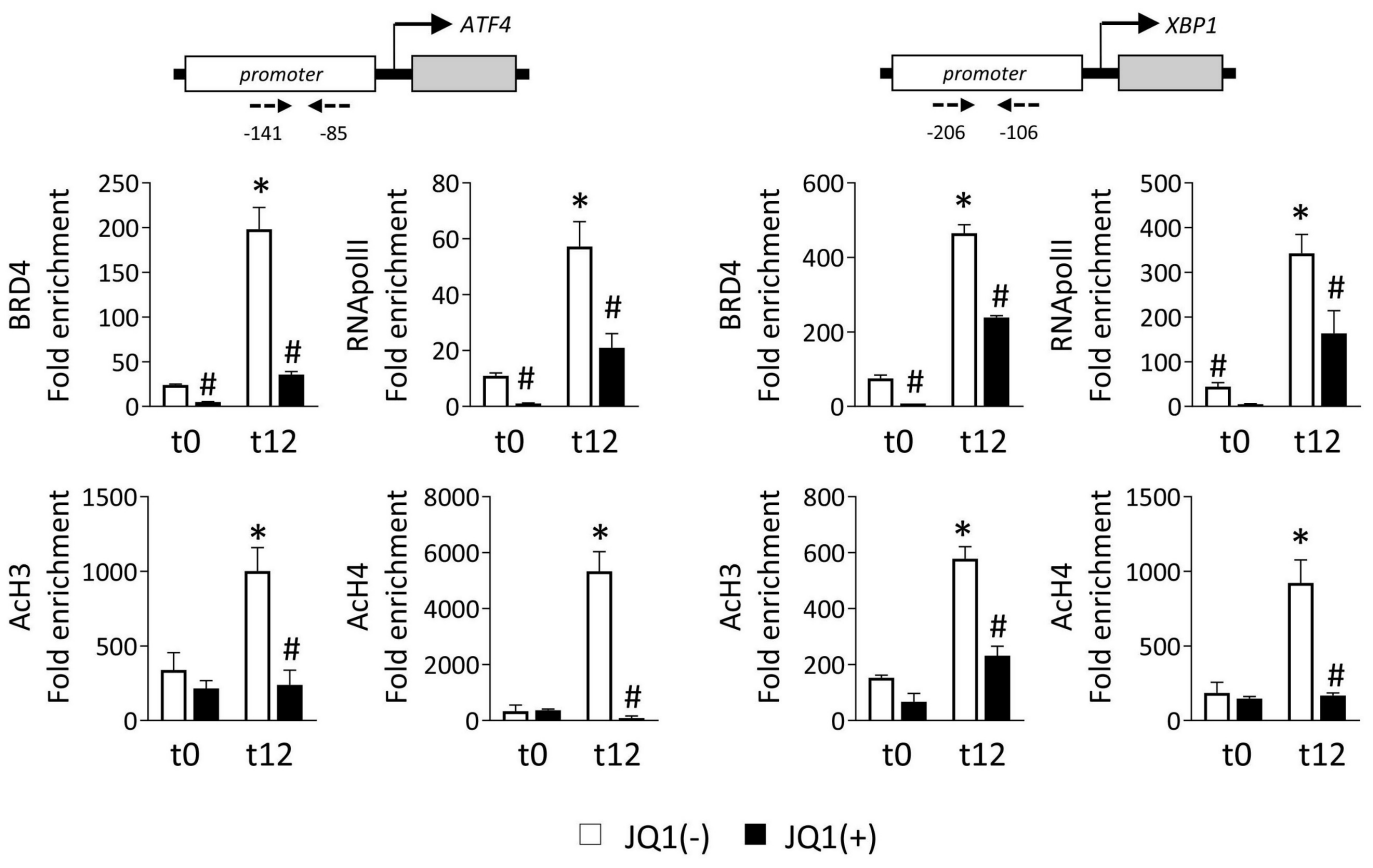
** JQ1 inhibits direct binding of BRD4 protein to UPR genes induced by hypoxia in HK-2 cells.** HK-2 cells were pretreated with or without JQ1 (+) or its enantiomer JQ1 (-) (500 nM, 24h), and subsequently cultured under conditions of normoxia (t0) and hypoxia (t12) at the times indicated. ChIP assays were performed with specific antibodies against BRD4, RNA POL II, AcH3 and AcH4, and the region of interest of *ATF4* (**A**) and *XBP1* (**B**) genes was amplified using specific primers (dashed arrows) by RT-PCR. Results are represented as the relative enrichment of each antibody relative to the IgG control. Data are expressed as the mean ± SEM of three independent experiments; *p<0.05 *vs.* t0 (normoxia) and # *vs.* cells treated with JQ1 (-).

BRD4 recognizes and binds to acetylated lysine of regulatory regions, thereby allowing the recruitment of the P-TEFb complex that phosphorylates RNA polymerase II (RNA pol II), and initiating gene transcription. To explore this, we performed ChIP assays with an anti-BRD4 antibody in HK-2 cells cultured under normoxia (t0) or hypoxia (t12) in the presence of the JQ1(+) inhibitor or its enantiomer, JQ1(-). Data show that in the presence of the nonactive control, JQ1(-), BRD4 is highly enriched in the promoter regions of the *ATF4* and *XBP1* genes after hypoxia (**Figure 3**). However, this recruitment was significantly reduced after treatment with JQ1(+). Similarly, the enrichment of the phosphorylated RNA pol II (Ser 2) observed under hypoxia in these genes was significantly reduced after blockage of the BRD4 binding by JQ1(+) (**Figure 3**). We also investigated whether enhancement of the levels of acetylated histones is the initial point for the recruitment of BRD4 to these genes. Acetylation levels in histone H3 (AcH3) and H4 (AcH4) were highly enriched in the promoter region of *ATF4* and *XBP1* genes under hypoxia, and thereby associated with their enhanced expression (**Figure 3**). Treatment with JQ1(+) reduced these acetylation levels at similar levels to observe under normoxia. Similar results were observed under Tg induction (**Figure S5A**).

Additionally, HK-2 cells induced with Tg were treated with DRB, a specific inhibitor of the component of the P-TEFb complex CDK9 (cyclin-dependent kinase 9). Results showed a dose-dependent decrease in ATF4 and XBP1 transcription levels (**Figure S5B**). Thus, we can suggest that recruitment of BRD4 and its binding to acetylated histones facilitate activation of the P-TEFb complex and, consequently, the phosphorylation of RNA pol II, which triggers the rapid expression of these UPR genes.

Moreover, consistent with our prior findings, BRD4 inhibition does not influence ATF6 expression. To corroborate this, the recruitment of BRD4 was analyzed along the *ATF6* promoter region, spanning approximately 900 bp and using H3 histone as a positive control. We observed that the recruitment of BRD4 was not enhanced in the promoter region of *ATF6* under hypoxic conditions, and consequently the treatment with the JQ1(+) inhibitor did not show any effect, reinforcing that BRD4 is not directly involved in the increased ATF6 expression after UPR activation (**Figure S6**).

### JQ1 reverts renal ischemia-mediated ER stress, ameliorating kidney function

**Figure 4:**
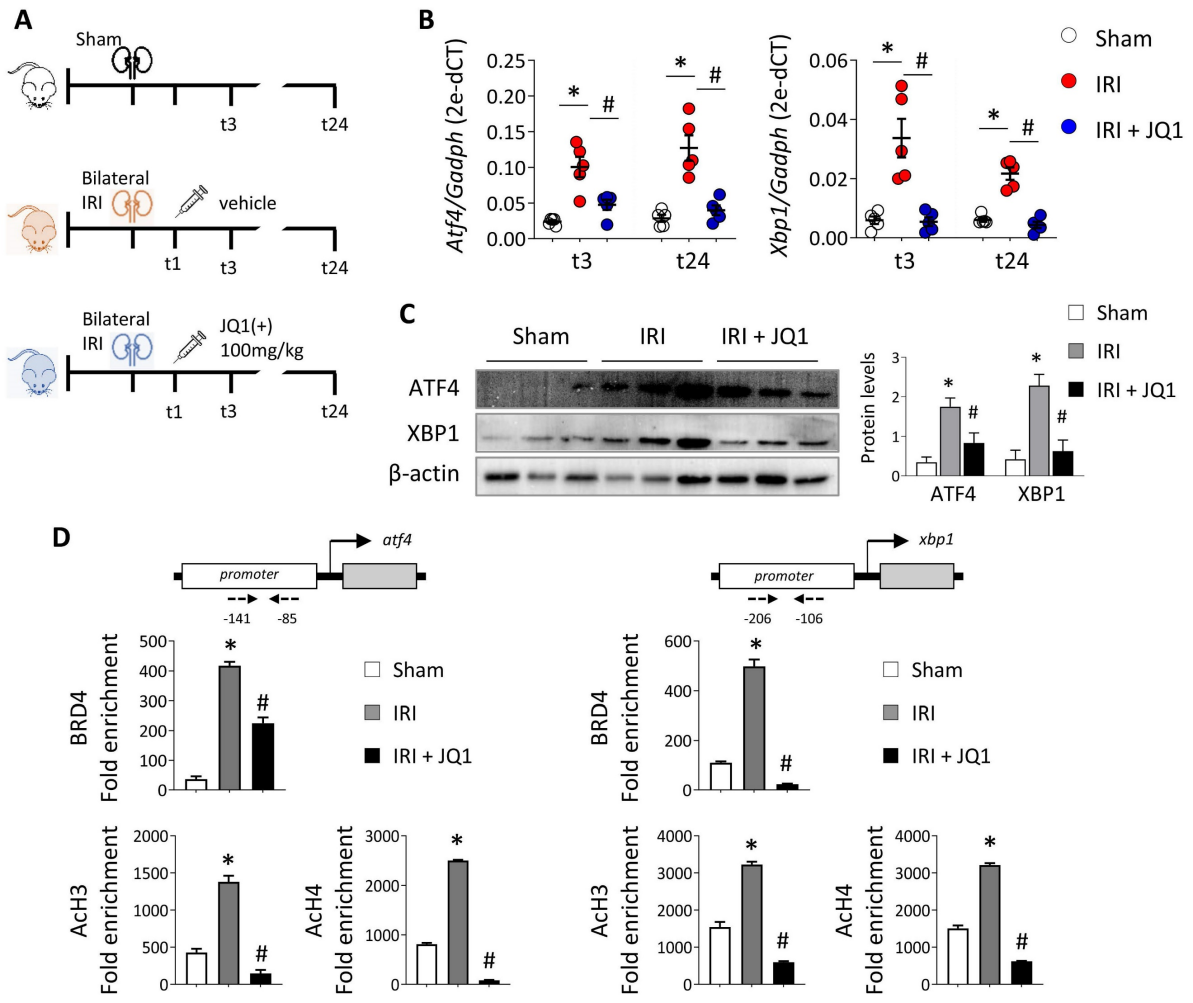
** Administration of JQ1 after IRI blocks BRD4 recruitment to UPR genes, decreasing their expression.** (**A**) C57BL/6 mice were treated with JQ1 (100 mg/kg) or vehicle (10% cyclodextran) 1 h after IRI. Sham mice were used as the control group. Samples were obtained at 3 h (t3) and 24 h (t24) post-ischemic damage. Groups: Sham, IRI (with vehicle) and IRI+JQ1 (administration of JQ1). (**B**) Gene expression levels were analyzed by RT-PCR at 3 h and 24 h. Results are means ± SEM of five animals per group. *p<0.05 *vs.* control and # *vs.* IRI group. (**C**) ATF4 and XBP1 protein levels were detected by western blot assay 3 h after IRI. (**D**) A ChIP assay was carried out in renal samples from the Sham, IRI and IRI+JQ1 groups using specific antibodies for BRD4, AcH3 and AcH4. Rabbit IgG was used as a negative control. Enrichment of binding regions in the *atf4* and *xbp1* promoters was quantified by RT-PCR using specific primers (dashed arrows). Data are summarized as the mean ± SEM of three independent experiments and expressed as the enrichment relative to the IgG negative control. Statistical analyses involved use of the two-tailed Student's unpaired t-test and the U-Mann Whitney test. *p<0.05 *vs.* Sham group; # p<0.05 *vs.* IRI group.

Next, the effect of JQ1 was evaluated in an *in vivo* model of renal damage induced by IRI. JQ1 (100 mg/kg) was administered intraperitoneally 1 h after injury to evaluate its therapeutic potential. Phenotypic and functional changes were analyzed at 3 h (t3) and 24 h (t24) after reperfusion (**Figure 4A**). The expression of *ATF4* and *XBP1* genes increased soon after damage remaining elevated even after 24 h (**Figure 4B**). Administering JQ1 impaired the overexpression of these genes, restoring similar levels to those in the sham group (**Figure 4B and 4C**).

We corroborated the recruitment of BRD4 to the regulatory region of these genes in the kidneys during IRI, associated with elevated levels of AcH3 and AcH4 (**Figure 4D**). However, the recruitment of BRD4 was significantly reverted in the kidneys of JQ1-treated mice and so the acetylated histone levels decreased at lower levels to that observed in the sham group. These results indicate that JQ1 acts to impair binding of BRD4 at the promoter region of these genes and, with it, the recruitment of the transcriptional machinery, while preventing their overexpression even in the presence of renal damage.

In line with our *in vitro* models, analysis of BRD4 levels showed a slight increase in BRD4 mRNA levels at 3h post-reperfusion, returning to control (sham) levels afterwards (**Figure S7**). Nevertheless, despite the substantial recruitment of BRD4 to the regulatory regions of *ATF4* and *XBP1*, there were no significant changes in BRD4 protein levels.

**Figure 5:**
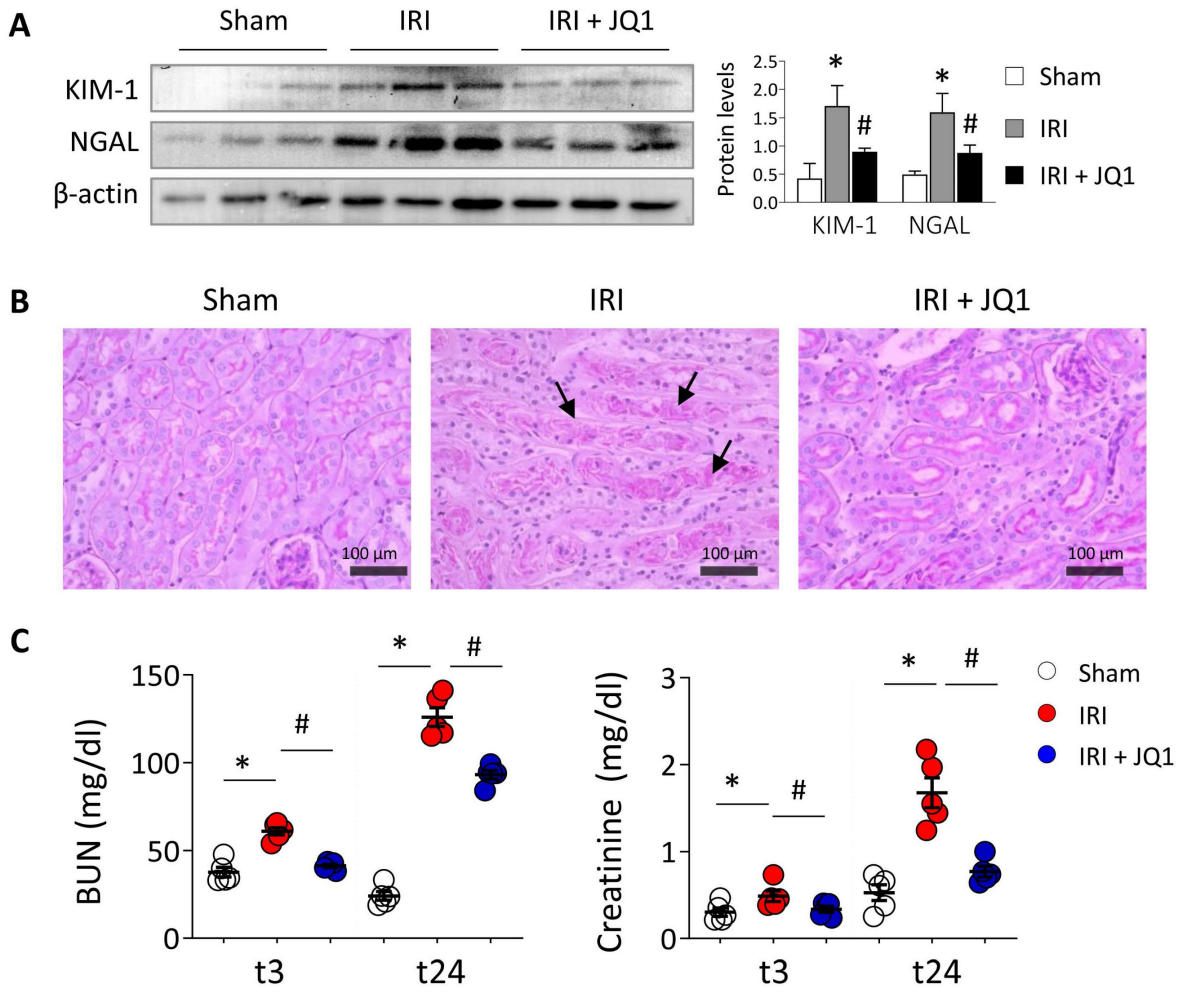
** Inhibition of BET proteins with JQ1 restores renal damage induced by *in vivo* IRI.** C57BL/6 mice were treated with JQ1 (100 mg/kg) or vehicle (10% cyclodextran) 1 h after IRI. Sham mice were used as the control group. Samples were obtained at 3 (t3) and 24 (t24) h post-ischemic damage. Groups: Sham, IRI (with vehicle) and IRI+JQ1 (administration of JQ1). (**A**) Renal KIM-1 and NGAL protein levels were analyzed by western blot 24 h post-damage. Data are summarized as the mean ± SEM of five animals per group. The western blot image shows the expression levels of three representative animals per group. Statistical analysis involved use of the two-tailed Student's unpaired t-test and the Mann-Whitney U test. *p<0.05 *vs.* Sham, and # *vs.* IRI group. (**B**) Representative PAS-stained sections for Sham, IRI and IRI+JQ1 groups. Scale bar= 100 µm and arrows indicate presence of tubular casts. **C**) Serum creatinine (mg/dl) and blood urea nitrogen (BUN, mg/dl) in mice from the three groups at 3 h (t3) and 24 h (t24) post-ischemic damage. The Mann-Whitney test was used. *p<0.05 *vs.* Sham and # *vs.* IRI group.

Additionally to these changes, JQ1 administration is associated to a reduced expression of the kidney injury markers, KIM-1 and NGAL (**Figure 5A**). PAS staining in the corticomedullary junction, the kidney region most susceptible to renal ischemic injury, shows that mice in the IRI group have tubular damage with loss of the tubular structure and epithelial cell connections as well as urinary cast within the tubule compared with the sham group (**Figure 5B**). However, mice treated with JQ1 had significantly fewer damaged tubules and, although some intratubular deposits were observed, the integrity and structure of the TECs was preserved (**Figure 5B**). Moreover, the significant enhancement of BUN and creatinine levels observed at 24 h in IRI mice was partially restored after JQ1 administration (**Figure 5C**). Thus, pharmacological blockage of ATF4 and XBP1 expression by JQ1 not only reduced UPR activation but also partially contributes to reduce the associated renal damage.

### BET proteins differentially regulate molecular pathways triggered by ATF4 and XBP1 under ER stress

Furthermore, we examined whether the administration of JQ1 could modulate the ATF4 and XBP1 downstream pathways in a similar way. For that, we generated stable knockout of *ATF4* and *XBP1* in HK-2 cells using CRISPR-Cas9 technology followed by transcriptomic analysis through RNA-seq. The complete absence of *ATF4* and *XBP1* before and after Tg-mediated ER stress induction was confirmed by sequencing (**Figure S8A**) and expression studies (**Figure S8B and S8C**).

**Figure 6:**
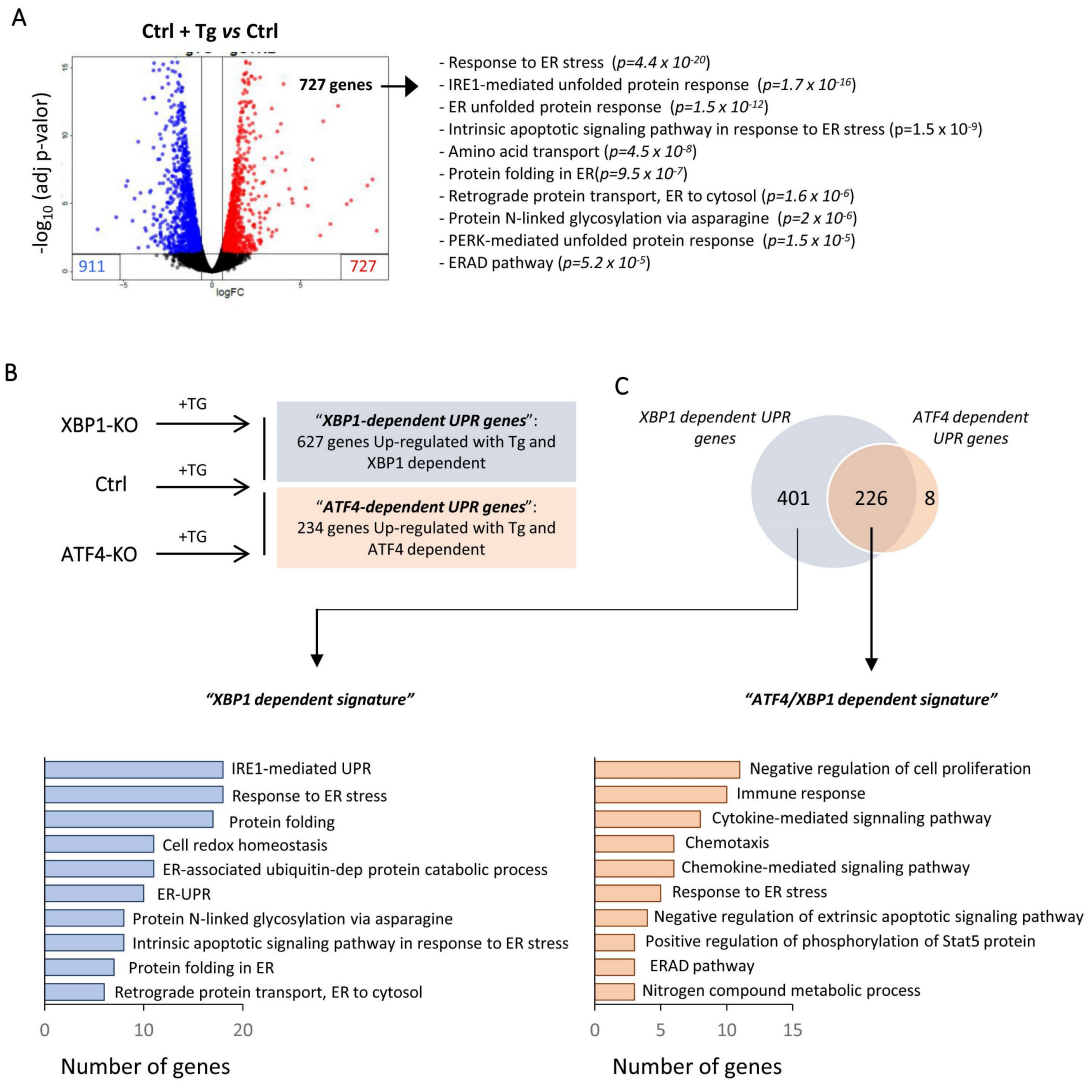
** XBP1 and ATF4-dependent transcriptional pathways following UPR activation in HK-2 cells.** Control HK-2 cells, and XBP1 (XBP1-KO) and ATF4 (ATF4-KO)-deficient cells were untreated or treated with Tg (4 μM, 24 h) before mRNA isolation and RNA sequencing analysis. (**A**) Volcano plot of genes differentially expressed between HK-2 cells treated with Tg relative to untreated cells. Upregulated and downregulated genes are shown in red and in blue, respectively. The ten most significant gene ontology (GO) pathways of upregulated genes after Tg treatment are shown, with the probability of each category in parentheses. (**B**) Scheme of the different HK-2 cell types (Ctrl, XBP1-KO, and ATF4-KO) treated with Tg and comparisons between them. “*XBP1-dependent UPR genes*” (blue) are derived from the genes upregulated in control (Ctrl) cells but not in XBP1 KO cells. “*ATF4-dependent UPR genes*” (red) are derived from the genes upregulated in control (Ctrl) cells but not in ATF4-KO cells. (**C**) Venn diagram of the comparison between “*XBP1-dependent UPR genes*” and “*ATF4-dependent UPR genes*” and GO analysis of the ten most significant categories of each one according to the number of genes.

Differential gene expression analysis revealed that under Tg induction of HK-2 cells, 727 and 911 genes were upregulated and downregulated, respectively (**Figure 6A and Table S3**). As expected, upregulated genes showed enrichment in biological processes mainly associated with “response to ER stress”, “protein folding in ER” or downstream pathways such as the “ERAD pathway” or “cell death” **(Table S3)**. To elucidate the upregulated genes by ATF4 and/or XBP1 under ER stress, we adopted a similar approach (UPR induction by Tg) using ATF4 (ATF4-KO) and XBP1 (XBP1-KO) knockout cells and making further comparison of upregulated genes under both conditions with control cells treated with Tg (**Figure 6B**). We identified 627 genes regulated by XBP1 and 234 ATF4-dependent genes. Surprisingly, we found that 96.6% (226/234) of the ATF4-dependent genes were also regulated by XBP1 and only eight genes (*SLC18B1*, *EFCAB6*, *CLDN11*, *MST1*, *AHRR*, *BBUF1*, *GPT2*, *TMEM184A*) were exclusively regulated by ATF4. Gene ontology (GO) analysis showed that the genes regulated only by XBP1 were associated with early processes induced under UPR activation, such as “protein folding”, “response to ER stress”, “IRE1-mediated UPR” and “protein folding”, among others (**Figure 6C and Table S4**). By contrast, ATF4 in collaboration with XBP1 (ATF4/XBP1) regulated mainly biological pathways related to “negative regulation of cell proliferation”, “immune response” or “cytokine and chemokine signaling pathways” (**Figure 6C and Table S4**). Thus, XBP1 mainly activates processes that can restore ER homeostasis, but also, in conjunction with ATF4, regulates pathological processes that perpetuate the damage.

**Figure 7:**
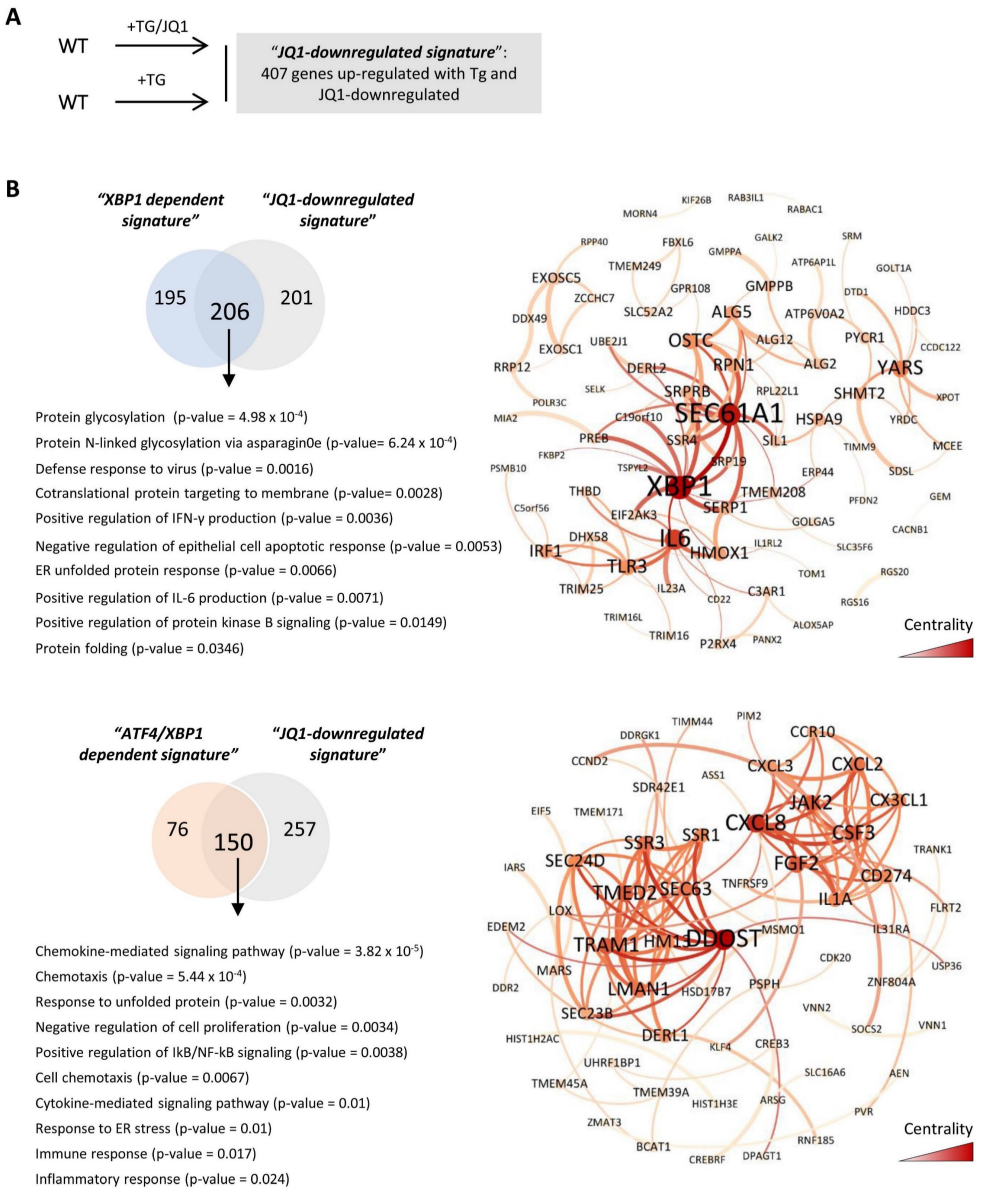
** JQ1 reverts expression of molecular pathways regulated by XBP1 and ATF4 under UPR activation.** (**A**) Scheme showing the comparative of HK-2 cells treated with Tg or the combination of Tg plus JQ1 to determine the signature of genes upregulated by Tg and modulated by JQ1, “*JQ1-dependent signature*”. (**B**) Venn diagrams showing comparisons between “*XBP1 dependent signature*” or “*ATF4/ XBP1 dependent signature*” and “*JQ1 dependent signature*” to select the 206 genes regulated by XBP1 and downmodulated by JQ1, and the 150 genes regulated by ATF4/XBP1 and downmodulated by JQ1. GO analysis of the ten most significant categories of genes whose expression was reduced by JQ1 under UPR activation and functional interaction networks of genes downmodulated by JQ1 and regulated by XBP1 or ATF4/XBP1. Network centrality is indicated by the color scale and node size.

To confirm the transcriptional signature induced under ER stress and modulated by the BETs inhibitor, HK-2 cells treated with Tg and JQ1(+) were analyzed by RNA-seq. We found 2515 genes differentially expressed relative to those in the control cells (979 upregulated, 1536 downregulated genes) (data not shown). Additionally, upregulated genes were compared with those in HK-2 cells treated only with Tg to identify the genes downmodulated by JQ1 under UPR activation (**Figure 7A**). We identified 407 genes whose expression, in the presence of JQ1(+), was not increased by Tg, and which were therefore regulated by BET proteins (**Table S5**). Using Venn diagrams and the previously determined XBP1- and ATF4/XBP1-dependent signatures, we found that 51.3% (206/401) of the XBP1-regulated genes and 66.3% (150/226) of the XBP1/ATF4-regulated genes were downmodulated by JQ1 (**Table S5**). Most significant pathways regulated exclusively by XBP1 and downmodulated by JQ1(+) were associated with “protein glycosylation” (*OSCT, ALG5*), the “defense response to virus” (*RPN1*), or “positive regulation of IFN-γ and IL-6 production” (*IRF1, TLR3, IL6, XBP1*) (**Figure 7B and Table S5**). However, pathways related to the immediate response mechanisms and involved in the degradation process of misfolding proteins aimed to restore the ER stress, such as “ERAD pathway” (*DERL3, OS9,*), “protein folding in ER” (*HSP90*/*GPR94* and *PDIA3*), “response to ER stress” (*ERN1*/IRE1A) or “response to unfolded proteins” *MANF*) were not regulated by JQ1(+) (**Figure S9 and Table S5).** These data were further confirmed in the *in vitro* and *in vivo* studies previously performed** (Figure S10A and S10B).**

**Figure 8:**
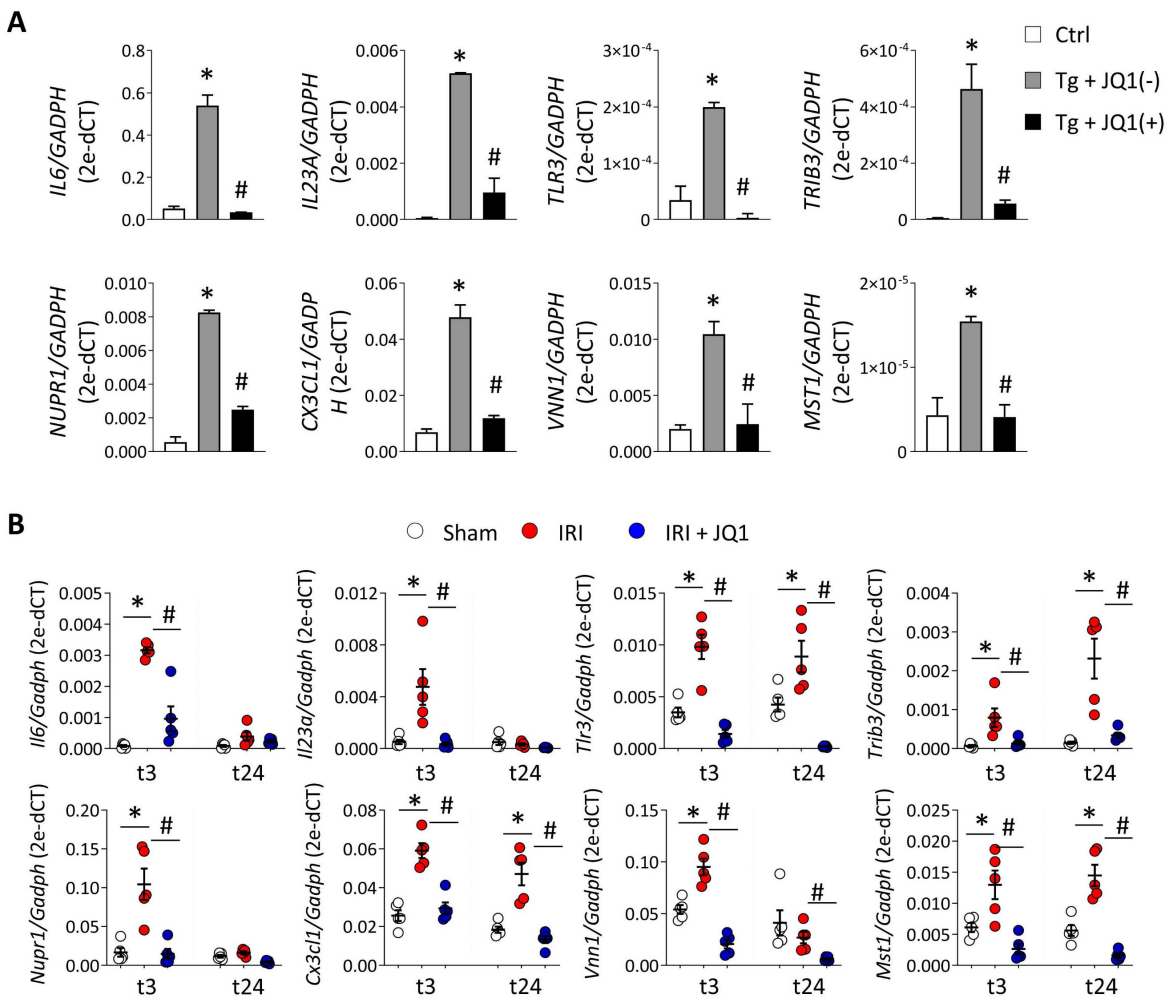
** Downregulation of immune, inflammatory and apoptotic genes as consequence of blockage of BET proteins under UPR activation.** (**A**) HK-2 cells were untreated or treated with Tg (4 μM, 24 h) in the presence of JQ1 (+) or its enantiomer, JQ1 (-). (**B**) Kidney samples were obtained from the sham, IRI and IRI+JQ1 mouse groups (n=5 per group) at 3 (t3) and 24 (t24) h post-reperfusion. Expression of XBP1-dependent genes (*IL6, IL23A, TLR3, TRIB3, NURP1*), XBP1/ATF4-dependent genes (*CXCL3, VNN1*) and ATF4-dependent genes (*MST1*) was measured by RT-PCR analysis; *GAPDH* was used as a housekeeping gene. Data are summarized as the mean ± SEM of at least three independent experiments. Statistical analyses involved use of the two-tailed Student's paired t-test and Mann-Whitney U test. *p<0.05 *vs.* control (Ctrl, DMSO) or sham group, and # *vs.* cells treated with Tg + JQ1 (-) or IRI group.

Additionally, ATF4/XBP1-regulated and BET-dependent genes were associated with immune and inflammatory responses (*IL1A, CD274, CSF3, VNN1*), chemotaxis and cytokine signaling pathways (*CXCL8, CXCL3, CX3CL1*), or the response to unfolded proteins (*TRAM1, DERL1*) (**Figure 7B and Table S5**). Some of these genes associated with the inflammatory response (*IL6, VNN1*), immune defense (*IL23, TLR3*) and cell death (*TRIB3, NUPR1, MST1*), and regulated by XBP1 (*IL6, IL23A, TLR3, TRIB3, NUPR1*), ATF4 (*MST1*) or both (*VNN1, CX3CL1*), were significantly reduced after JQ1(+) treatment in the *in vitro* and *in vivo* models previously established (**Figure 8A and 8B**). These results demonstrate that inhibition of BET proteins impairs the transcriptional programs associated with the renal damage triggered by persistent UPR activation, without affecting the initial mechanisms of the ER stress response, thus enabling early repair.

## Discussion

BET proteins are involved in many inflammatory and pro-fibrotic processes triggered during chronic kidney damage. However, their role in the initial stages after acute kidney injury is poorly understood and some apparently contradictory observations have been reported. Here, we demonstrate that pharmacological inhibition of BET proteins blocks the ATF4 and XBP1-mediated maladaptive UPR pathway that is activated under sustained ER stress and is triggered by ischemic renal injury.

Numerous studies had previously suggested that disturbance of ER proteostasis and thereby activation of the UPR pathway is involved in several kidney-associated pathologies 29-31. Therefore, modulation of the balance between adaptive and maladaptive UPR activation represents a crucial therapeutic strategy for preventing or attenuating the progression of renal damage 32. However, the involvement of epigenetic mechanisms in regulating and modulating the UPR pathway has not been thoroughly explored. Our group recently reported that the methylation dynamics of H3K9 and H3K27 histones are key to modulating expression of the ATF4 and XBP1 transcription factors under ER stress conditions in tubular cells 33.

BRD4 belongs to a family of transcriptional mediators, the BET proteins, that recognize and bind to acetylated residues to further recruit the p-TEFb elongation complex and activate RNA pol II, leading to gene transcription 34. Our results reveal that in tubular cells exposed to hypoxia, BRD4 is highly recruited to the promoter region of *ATF4* and *XBP1* genes, a process abolished in the presence of JQ1. The reduction in the BRD4 recruitment is correlated with an impaired RNA pol II phosphorylation, leading to the inhibited transcription of these genes. BRD4 also binds to enhancer regions, regulating the expression of genes requiring a quick response, such as those mediated by NF-κB or the proto-oncogene c-MYC 35, 36. The activation of UPR after an ischemic insult needs to be immediately triggered in the cell if the damage is to be repaired and if ER homeostasis is to recover. Wilflingseder *et al.* 37 recently demonstrated that, after ischemic renal damage, BRD4 is located next to the acetylation of H3K27 residues in “enhancer” and "super-enhancer" regions, allowing rapid enhancement of gene transcription in response to the initial stimulus. These data reinforce our results by revealing the key role of BET proteins in the rapid and efficient response following to an ischemic damage. Moreover, these authors demonstrated that the BRD4 expression in the renal tissue is not modified after ischemia, in accordance with our results where the BRD4 protein levels remained unchanged, both* in vitro* and *in vivo*, following UPR pathway activation. Thus, the changes induced by BRD4 after an ischemic insult are mainly mediated by the recruitment of this epigenetic remodeler to the regulatory regions of the *ATF4* and *XBP1* genes. However, our results differ from those published by Liu *et al.* 38 who reported a significant increase in BRD4 protein levels during hypoxia and subsequent reoxygenation which were then reduced in the presence of JQ1. Their study concluded that the contribution of JQ1 to alleviate the ischemic renal damage is attributed to the modulation of BRD4 expression, but in any case, they demonstrated the changes in the recruitment of BRD4 to the analyzed genes. Moreover, their animal model of IRI, involving right kidney nephrectomy and 30 min of ischemia in the left kidney, differ from us, so these results should be analyzed with caution.

In our *in vitro* cellular context, ATF6 expression was induced after UPR activation, which was not attenuated by JQ1 or specific BRD4 blockage. Our data indicate that BRD4 is recruited to the regulatory region of determined genes contributing to modulate their expression. So, the treatment with JQ1 will affect only to the expression of specific genes without global alterations, as evidenced by the unchanged ATF6 protein levels that were detected. The contribution of the three different pathways of the UPR to the renal damage is a complex process, because of their interplay 14. In fact, we repot that in absence of ATF4, the expression of XBP1 is reduced and both are modulating jointly the expression of several genes. However, neither ATF4 nor XBP1 modulates the expression of ATF6. Thus, it will be the final balance between the adaptive and maladaptive pathways that ultimately dictates the progression of the damage.

Until now, some studies have proposed administration of JQ1 as a possible treatment against AKI induced by IRI, but results remain contradictory. Liu *et al.* 38 showed that 7 days pre-treatment with JQ1 in an I/R mouse model prevents apoptosis mediated by ROS production, leading to the maintenance of tubular morphology, which favors survival. Likewise, 7 days treatment with MS417, another specific BRD4 inhibitor, followed by unilateral IRI, could attenuate neutrophil infiltration in the kidney, which is associated with less tubular damage and the reduction of KIM-1 and NGAL in the face of early damage 18. Consistent with these reports, we found that administering JQ1 reduced expression of the neutrophil chemoattractants, CXCL8 (IL8) and CXCL2 (MIP2b), which could be associated with the observed in the lower levels of KIM-1 and NGAL and, eventually, functional recovery. Nevertheless, it has been reported that administering JQ1 at the time of ischemic damage not only fails to alter renal function but also produces higher mortality 37. However, the progressive delay of JQ1 administration after the ischemia event, has different molecular effects and reduces systemic toxicity. In our experience, administering JQ1 only 1 h after the damage does not affect survival one day after the ischemic insult. Consistent with our results, Wilflingseder *et al.* 37 reported that surviving mice in the JQ1-treated group had the lowest creatinine value on day 1 post-surgery, indicating recovery of renal function in 20% of mice treated at the time of surgery. It is important to note that the effect of the treatment may depend on the intensity of the damage because the ischemia we performed was more severe and the molecular mechanisms of damage response activated could have differed. In another pathological context, administration of BRD4 inhibitors, 2 or 4 h post-ischemic stroke, protected against brain damage by reducing inflammation, oxidative stress and the activation of NLRP3 inflammasome 39, 40. More studies are needed to determine the optimal timing for administering BET inhibitors after IR kidney injury.

The role of XBP1 differs with the time since injury and the cellular context. In initial stages, XBP1 is essential for cell adaptation and restoring ER homeostasis, but sustained and aggravated UPR activation triggers oxidative stress and an inflammatory response, aggravating the loss of renal function 41-43. However, overexpression of ATF4 during renal pathology is mainly associated with development and progression of the disease 44-47. Accordingly, our data revealed that 96.6% of ATF4-dependent genes are also regulated by XBP1. It is known that ATF6 and XBP1 form heterodimers to induce the expression of the main components of the ERAD response 48, 49, but it is the first time, to our knowledge, that ATF4 is proposed to regulate a coordinated response with XBP1.

Apoptosis and inflammation are essential to the pathogenesis of a wide variety of renal diseases and to the AKI-to-CKD transition 50, 51. Among UPR-induced genes and JQ1-downregulated genes, *TRIB3* and *NUPR1* have been shown to reduce apoptosis, favoring autophagy and cell survival 52-54. Additionally, blockage of IL6 and TLR3 signaling reduces inflammation, and inhibition of the IL23-Th17 axis might reduce the infiltration of T lymphocytes 55, 56. Others, such as *CXCL2*, *CX3CL1*, *IL-1A* and *VNN1*, had already been proposed as targets for alleviating renal damage 57-59. We also observed downregulation of the *DDOST* gene by JQ1, which is involved in oxidative stress and chronic inflammation in the kidney 60.

It is worth emphasizing that JQ1 does not inhibit the adaptive pathways mediated by ATF4 and XBP1, aimed to restore ER homeostasis. Chaperones involved in degrading misfolding proteins and cell survival pathways (GRP94), molecules key to promote the re-folding of misfolded proteins (PDIA3) and genes such as *DERL3* and *OS9* associated to ERAD protective mechanism remain unmodified. Likewise, the expression of genes such as *MANF*, which is a cytoprotective gene with anti-inflammatory and anti-oxidative properties in kidney damage, is not modulated by JQ1 61-64. It has been previously reported that genes such as *HSPA5* or *PDIA3* are only partially regulated by XBP1, and ATF6 (not modulated by JQ1) could be regulating them 65, 66. Likewise, we cannot obvious that JQ1 could be involved in the regulation of numerous genes that in addition to UPR pathway contribute to the renal damage as it has been previously reported by our group among others 19-22, 37. Additional studies using ATF4 and XBP1 knockout mice in tubular cells will be required to analyze the specific involvement of these genes in AKI.

In conclusion, our findings identify BRD4 as a key regulator of overexpression of the ATF4 and XBP1 UPR genes after acute kidney injury induced by IRI. Administration of the potent BET protein inhibitor, JQ1, soon after damage might tip the balance between the adaptive and maladaptive UPR pathways. JQ1 could block the molecular pathways related to renal damage such as inflammation, oxidative stress and cell death, while preserve the mechanisms that restores ER homeostasis.

## Supplementary Material

Supplementary figures and tables 1-2, table legends for tables 3-5.

Supplementary table 3.

Supplementary table 4.

Supplementary table 5.

## Abbreviations

BET: bromodomain and extra-terminal
CKD: chronic kidney disease
ER: endoplasmic reticulum
IRI: Ischemia reperfusion injury
KO: knockout
TECs: tubular epithelial cells
Tg: Thapsigargin
UPR: Unfolded protein response
