# Supplementary material for: Inhibition of BRD4 Attenuates ER Stress-induced Renal Ischemic-Reperfusion Injury
Source: Int J Biol Sci. 2024 Feb 11;20(5):1547–62. doi: 10.7150/ijbs.83040 (PMC10929187; doi:10.7150/ijbs.83040)
Supplement: Supplementary file 1 — Supplementary figures and tables 1-2, table legends for tables 3-5. [file ijbsv20p1547s1.pdf]

SUPPLEMENTARY MATERIAL

Figure S1. Pharmacological treatment and silencing of BRD4 downregulates ATF4 and XBP1 UPR genes in tubular epithelial cells (TECs).

Figure S2. Hypoxia induces the expression of UPR genes.

Figure S3. Treatment with JQ1 blocks ATF4 and XBP1 expression under hypoxia/reoxygenation conditions.

Figure S4. BRD4 expression remains unchanged after UPR induction in HK-2 cells.

Figure S5. JQ1 inhibits the direct binding of BRD4 protein to UPR genes induced by Tg in tubular renal cells.

Figure S6. BRD4 is not involved in the gene transcription of ATF6 induced by hypoxia in HK-2 cells.

Figure S7. BRD4 expression remains stable in mice with IRI.

Figure S8. CRISPR interference induced specific and irreversible gene silencing of XBP-1 and ATF4 in HK-2 cells.

Figure S9. Treatment with JQ1 avoids the downmodulation of the adaptive ER stress pathways.

Figure S10. Pathways associated with restoring ER homeostasis were not modulated by JQ1 treatment.

Table S1. Primers sequences for RT-PCR and ChIP assay in human and mouse genes.

Table S2. Gene-targeted sequences and primers for CRISPR/Cas9 mediated knockout studies.

Table S3. Genes upregulated and downregulated in HK-2 cells after Tg induction, and Gene Ontology (GO) categories of upregulated genes.

Table S4.- Genes and GO categories from the "*XBP1-dependent signature*" and "*ATF4/XBP1-dependent signature*".

Table S5.- Genes and GO categories from the "*JQ1-dependent signature*", "*JQ1-dependent XBP1 signature*" and "*JQ1-dependent ATF4/XBP1 signature*".

Figure S1. Pharmacological treatment and silencing of BRD4 downregulates *ATF4* and *XBP1* UPR genes in tubular epithelial cells (TECs). Renal TECs (HK-2 cell line) were treated with DMSO (Ctrl) or Tg (4 µM, 24 h) in the absence or presence of different doses of the JQ1 (+) or its enantiomer JQ1(-) (**A**), and I-BET 762 **(B)** inhibitors for 24 h. Gene expression was analyzed by RT-PCR analysis, with *GAPDH* used as a housekeeping gene. Data are summarized as the mean ± SEM of at least three independent experiments. Two-tailed Student's paired t-tests were used; *$p < 0.05$ *vs.* control; # $p < 0.05$ *vs.* Tg+ JQ1(-)-treated cells. (**C, D**) HK-2 cells were transfected with a specific siRNA against BRD4 (siRNA BRD4, 25 and 40 nM) or control siRNA (siRNA Ctrl, 40 nM) for 48 h and followed by Tg treatment (4 µM, 24 h). Transcriptional levels of *BRD4*, *ATF4* and *XBP1* were determined by RT-PCR (**C**), and protein levels (**D**) were assayed by western blot. Data are summarized as the mean ± SEM of three independent experiments. Statistical analyses involved the two-tailed Student's paired t-test and the Wilcoxon test. *$p < 0.05$ *vs.* Ctrl and # *vs.* control siRNA Ctrl-treated cells.

Figure S2. Hypoxia induces the expression of UPR genes. HK-2 cells were exposed to normoxia (t0) or different hypoxia times, 1 (t1), 3 (t3), 6 (t6) and 12 (t12) hours. Transcriptional levels of, *ATF4*, *XBP1* and *ATF6* were determined by RT-PCR. Data are expressed as mean ± SEM of three independent experiments. Two-tailed Student's paired t-test and Wilcoxon test analysis were used. *$p < 0.05$ vs t0 (normoxia).

Figure S3. Treatment with JQ1 blocks ATF4 and XBP1 expression under hypoxia/reoxygenation conditions. HK-2 cells were subjected to 12 h of hypoxia (t12) followed by various reoxygenation time points (2h, 4h, 6h H/R time) in the absence or presence of JQ1(+) or its enantiomer JQ1(-). Gene expression was analyzed by RT-PCR analysis (**A**) and protein levels (**B**) were analyzed by western blot. Data are expressed as the mean ± SEM of at least three independent experiments. *GADPH* and β-actin were used as housekeeping markers of RT-PCR and western blot, respectively. Statistical analyses involved use of the two-tailed Student's paired t-test and the Wilcoxon test. *$p<0.05$ *vs*. t0 (normoxia), # *vs.* cells in hypoxia (t12) treated with JQ1(-) and $ *vs*. cells under H/R times treated with JQ1 (-).

Figure S4. BRD4 expression remains unchanged after UPR induction in HK-2 cells. HK-2 cells were cultured under different experimental conditions; (**A**) with DMSO (Ctrl) or Tg (4 µM, 24 h), (**B**) exposed to normoxia (t0) or hypoxia (t12) conditions and (**C**) exposed to normoxia (t0) or hypoxia (t12) conditions followed by different reoxygenation time, including 2h, 4h and 6h (H/R time). Transcriptional levels of *BRD4* were determined by RT-PCR (left panel) and data are expressed as mean ± SEM of at least three independent experiments. Protein levels were assayed by western blot (middle and right panel). A representative image is showed in the middle panel are data from two or three independent experiments (marked as #) are showed in the right panel. Data *Gapdh* and β-actin were used as loading controls. Two-tailed Student's paired t-test was used. *p<0.05 *vs*. Ctrl cells.

Figure S5. JQ1 inhibits the direct binding of BRD4 protein to UPR genes induced by Tg in tubular renal cells. (**A**) HK-2 cells were cultured with Tg (4 µM, 24 h) in the presence of JQ1(+) or its enantiomer JQ1(-) (500 nM, 24h). ChIP assays were performed with specific antibodies against BRD4, RNA POL II, AcH3 and AcH4, and the region of interest of each gene was amplified by RT-PCR using specific primers (dashed arrows). The results are represented as the relative enrichment of each antibody versus IgG control. Data are expressed as the mean ± SEM of three independent experiments; *p< 0.05 vs Ctrl cells and # vs cells treated with Tg+ JQ1(-). (**B**) HK-2 cells were treated with Tg (4 µM, 24 h) and DRB (20-60 µM) was added during the last 6 h of culture. Gene expression was analyzed RT- PCR. Data are expressed as mean ± SEM of three independent experiments. Two-tailed Student's paired t-test was used. *p < 0.05 vs Ctrl; # p< 0.05 vs Tg-treated cells.

Figure S6. BRD4 is not involved in the gene transcription of *ATF6* induced by hypoxia in HK-2 cells. HK-2 cells were treated with or without JQ1(+) or its enantiomer JQ1(-) (500 nM, 24 h) before exposure to normoxia (t0) or hypoxia (t12) conditions and relative enrichment of BRD4 and Histone 3 (H3) were analyzed in five different regions (R1-R5) of the *ATF6*promoter by RT-PCR using specific primers (dashed arrows). The results are represented as the relative enrichment of each antibody versus IgG control. Data are

expressed as the mean ± SEM of three independent experiments and two-tailed Student's paired t-test was used.

Figure S7. BRD4 expression remains stable in mice with IRI. Mice were subjected to renal bilateral ischaemic injury for 45 min and sacrificed at 3 h, 6 h, 12 h and 24 h. Sham mice were used as the control group. Expression levels of BRD4 were quantified by RT-PCR (n=6-7 mice per group) and protein levels were assayed by western blot (n=3-4 mice per group). Data are represented as mean ± SEM. *Gapdh* and β-actin were used as controls. Statistical analyses involved use of Mann-Whitney test. *$p < 0.05$ vs Sham.

Figure S8. CRSPR interference induced specific and irreversible gene silencing of XBP-1 and ATF4 in HK-2 cells. (**A**) Genomic DNA was extracted from HK-2 cells transfected with the empty vector (HK-2-WT), and HK-2 cells silenced for XBP1 (XBP1-KO) and ATF4 (ATF4-KO). Sanger sequencing was performed to show the deletion of 8 and 7 nucleotides (nt) in *ATF4* (exon 2) and *XBP1* (exon 4) genes, respectively. Transcriptional expression (**B**) and protein levels (**C**) of ATF4, XBP1 and ATF6 in control cells (HK-2-WT) and after silencing of *XBP1* (XBP1-KO) and *ATF4* (ATF4-KO) genes. Expression levels are summarized as the mean ± SEM of three independent experiments. The two-tailed Student's paired t-test and the Wilcoson test was used. *$p< 0.05$ *vs.* control; # $p< 0.05$ *vs.* expression in HK-2-WT cells.

Figure S9. Treatment with JQ1 avoids the downmodulation of the adaptive ER stress pathways. Venn diagrams showing the comparisons between "*XBP1 dependent signature*" or "*ATF4/ XBP1 dependent signature*" and "*JQ1 dependent signature*" to select the genes regulated by XBP1 but not modified by JQ1, 195 genes; and the genes regulated by ATF4/XBP1 and unchanged after JQ1 treatment, 76 genes. GO analysis of ten most significant categories of genes decreased by JQ1 under UPR activation and functional interaction networks of genes unmodified by JQ1 and regulated by XBP1 or ATF4 /XBP1. Network centrality is indicated by the color scale and node size.

Figure S10. Pathways associated with restoring ER homeostasis were not modulated by JQ1 treatment. (**A**) HK-2 cells were treated with Tg (4 µM, 24 h) in the presence of

JQ1(+) or its enantiomer, JQ1(-). (**B**) Kidney samples obtained from the Sham, IRI and IRI+JQ1 mouse groups (n=5 mice per group) at 3 (t3) and 24 (t24) h post-reperfusion. *Gapdh* was used as a control housekeeping gene. *In vitro* data are summarized as the mean ± SEM of at least three independent experiments. Statistical analyses involved use of the two-tailed Student's paired t-test and Mann-Whitney U test. *p<0.05 *vs.* control (DMSO) or Sham group.

Table S1. Primers sequences for RT-PCR and ChIP assay in human and mouse genes.

	Forward (5´-3´)	Reverse (5´-3´)
**Human RT-PCR**		
ATF4	CTTGATGTCCCCCTTCGACC	CTTGTCGCTGGAGAACCCAT
ATF6	GCTGCAATTGGAAGCAGCAA	ACCGAGGAGACGAGACTGAA
BRD4	CCCCTCGTGGTGGTGAAG	GCTCGCTGCGGATGATG
CX3CL1	CACCACGGTGTGACGAAATG	TCTCCAAGATGATTGCGCGT
DERL3	CCTCAGCCCCTTTCAACTCT	GAAGTTGGTGACGAGCCTCC
ERP57	GCTAGAACTCACGGACGACA	TCAGGGTTGGATATCCACTG
GAPDH	TGCCATGGGTGGAATCATATTGGA	TCGGAGTCAACGGATTTGGTCGT
GRP94	CTTCCAAGCCGAAGTTAACAGA	GCATTTGAAATCAGTTCTCTCAGGA
IL23A	GACCCACAAGGACTCAAGGAC	ATGGGGCTATCAGGGAGTAGAG
IRE1a	CGATGGACTGGTGGTAACTG	GTTGATGTGCACCACCTTTC
MANF	TATAAAGTTCTGCCGGGAAGC	GATTTTGGTGGCTGCATCATC
MST1	ATACCATGGCCAAGCGGAAT	TCAGCATAAGGGGGCTTTCC
NUPR1	CCCACTTCACCTCTGACTCC	GGTCACCAGTTTCCTCTCGT
OS9	GGACGCCACATCCAGCAATA	CCAGTCGAAGGCTGATTGGT
TLR3	AGATTACCAGCCGCCAACTT	GCTCATTGTGCTGGAGGTTC
TRIB3	GAGATACTCAGCTCACGGGC	ATCTTGCCGAAGAGCAGGAC
VNN1	GATATTGCCCAATGCCACCC	TATGCGCACCCTGATCTGC
XBP1s	GCTGAGTCCGCAGCAGGT	CTGGGTCCAAGTTGTCCAGAAT
**Human assay**		
GAPDH (VIC) Hs02758991_g1		
IL6 (FAM*)* Hs00174131_m1		
**Human ChIP**		
ATF4	GTTGGCATGAAGCCCTCTTGAATAA	AGAGTGCTGTAGCTGTGTGTTC
ATF6-R1	ACGTGGTCCTAGAAGCATACG	TCCATTTTAAATTTCAGCGGCCA
ATF6-R2	TTTCAGTTGGAGTTCGTGATGT	AAGTCCAAGCGAGTCTACCC
ATF6-R3	CTTACCTCGGTTACTGTCCCAG	ACGCGTGTATGAAAGAAAAACACTA
ATF6-R4	TTGTTCTGAGATAGCCACGC	AAAGCCCCTTTCTGAACATTATGG
ATF6-R5	TCCCCATAAAACAGCGGGAC	GCACGAGGGATTTGTACGAC
XBP1	TCTCGATATGTGATGGTGTGTCC	CCAACTGAAGTGAGCCTAACG
**Mouse RT-PCR**		
atf4	GCAGCAGCACCAGGCTCT	TTGTCCGTACAGCAACACTG
atf6	CTTCCTCCAGTTGCTCCATC	CAACTCCTCAGGAACGTGCT
brd4	TGCTCAGGAATGTATCCAGGAC	AGACGATGTCATCTCCAGGC
cx3cl1	TGCGACAAGATGACCTCACG	CATTGTCCACCCGCTTCTCA
derl3	GCAAGGCTGACTTCGTTTTC	GCCTGTCCCAGGAAAAACAG
erp57	GTGTGGACATTGCAAGAGGC	TTGGCAGTGCAATCCACCTT
gapdh	GAAGGTCGGTGTGAACGGA	GTTAGTGGGGTCTCGCTCCT
grp94	TGTGTCCTGCTGACCTTCGG	TTTACCCAGGTCCTCTTCCACT
il23a	TGCTGGATTGCAGAGCAGTAA	TTCATATGTCCCGCTGGTGC
ire1a	GTCCCAACACACGTGGAAGA	AGTTTCGTCAGGCCTTCGTT
manf	AAGTTTTGCCGTGAAGCAAGA	GATGATCTTGGTGGCAGCATC
mst1	TGACAGCCCTCACGTAGTCA	AACGTCTTGTTCCGTAGCCG
nupr1	CACCAACAGCCAACCCTTCC	CTCTCTTGGTCCGACCTTTCC
os9	ATGCCCCTTGTTTGCTGAAG	CCTTTGATCTCCGAGTCTTCCA
tlr3	TACAAAGTTGGGAACGGGGG	GGTTCAGTTGGGCGTTGTTC
trib3	TGCCAAGTGTCCAGTCCTAA	CAGCAGGTGACAAGTCTGAGG
vnn1	ATGGCATATACGGTGTGCGT	CAGTGAGAGTCGCTGGTGTT
xbp1s	GAGTCCGCAGCAGGTG	GTGTCAGAGTCCATGGGA
**Mouse assay**		
Gapdh (VIC) Mm99999915_g1		

Il6 (FAM) Mm00446190_m1		
**Mouse ChIP**		
atf4	GAGATGGCAGGTGTGACAGT	CTATTGGCTCTGCACCTGGG
xbp1	ACATGCTAGCCAAGGCTCTAGT	GCAAAACTAAATGTAGCAGGGTAGT

Table S2. Gene-targeted sequences and primers for CRISPR/Cas9 mediated knockout studies.

Gene	Targeted exon	Primer sequence (5´-3´)
ATF4	2	Targeted sequence: TCTCTTAGATGATTACCTGGAGG<br>Forward: CACCGTCTCTTAGATGATTACCTGG<br>Reverse: AAACCCAGGTAATCATCTAAGAGAC
XBP1	4	Targeted sequence: CACCGGTCAATACCGCCAGAATCCA<br>Forward: CACCGGTCAATACCGCCAGAATCCA<br>Reverse: AAACTGGATTCTGGCGGTATTGACC

Table S3 (xls file). Genes upregulated and downregulated in HK-2 cells after Tg induction, and Gene Ontology (GO) categories of upregulated genes.

Table S4 (xls file). Genes and GO categories from the "*XBP1-dependent signature*" and "*ATF4/XBP1-dependent signature*".

Table S5 (xls file). Genes and GO categories from the "JQ1-dependent signature", "*JQ1-dependent XBP1 signature*" and "*JQ1-dependent ATF4/XBP1 signature*".

Figure S1

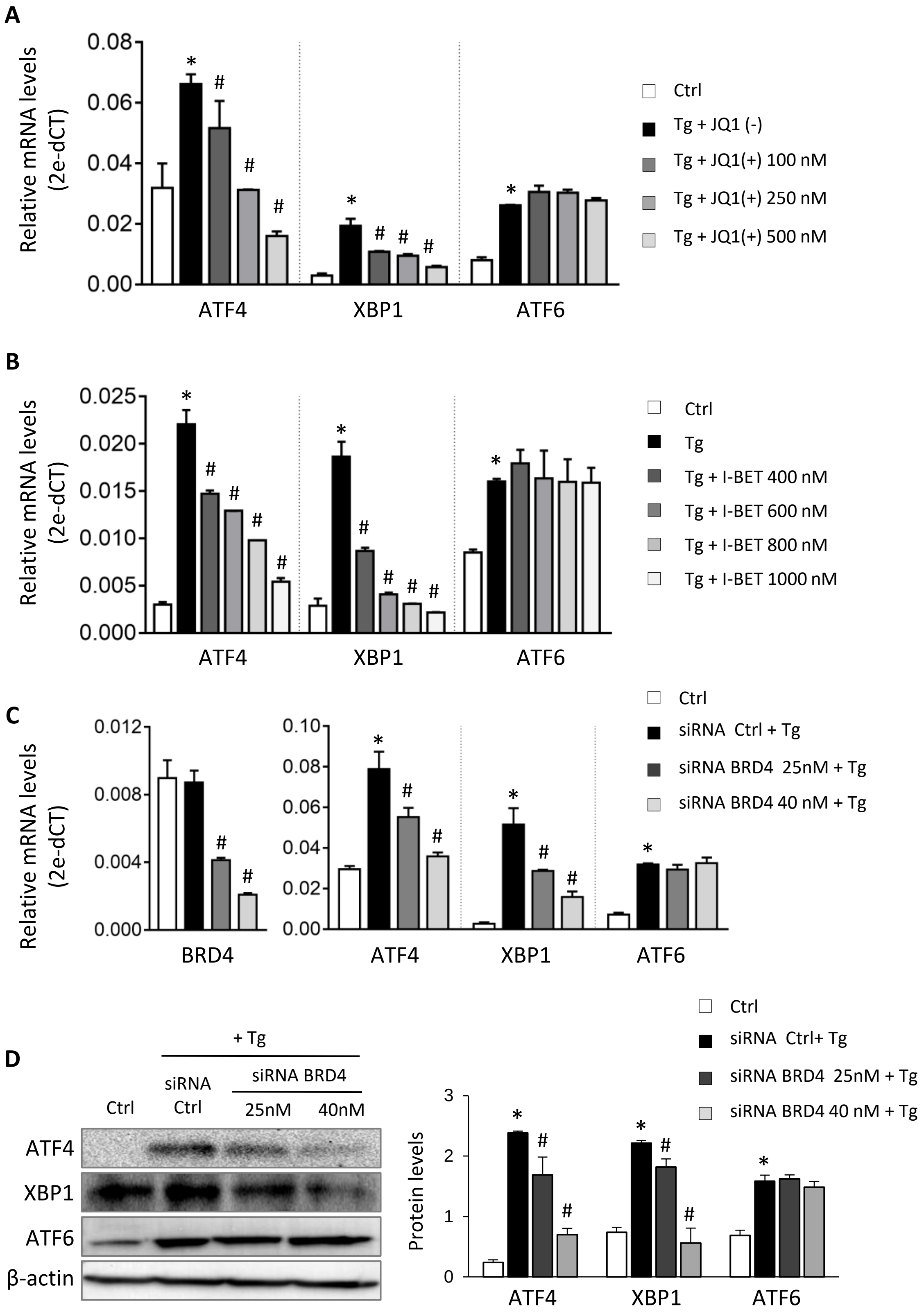
A
Relative mRNA levels (2e-dCT)
0.08
0.06
0.04
0.02
0.00
ATF4
XBP1
ATF6
Ctrl
Tg + JQ1 (-)
Tg + JQ1(+) 100 nM
Tg + JQ1(+) 250 nM
Tg + JQ1(+) 500 nM
B
Relative mRNA levels (2e-dCT)
0.025
0.020
0.015
0.010
0.005
0.000
ATF4
XBP1
ATF6
Ctrl
Tg
Tg + I-BET 400 nM
Tg + I-BET 600 nM
Tg + I-BET 800 nM
Tg + I-BET 1000 nM
C
Relative mRNA levels (2e-dCT)
0.012
0.008
0.004
0.000
BRD4
0.10
0.08
0.06
0.04
0.02
0.00
ATF4
XBP1
ATF6
Ctrl
siRNA Ctrl + Tg
siRNA BRD4 25nM + Tg
siRNA BRD4 40 nM + Tg
D
+ Tg
Ctrl
siRNA Ctrl
siRNA BRD4
25nM
40nM
ATF4
XBP1
ATF6
β-actin
Protein levels
3
2
1
0
ATF4
XBP1
ATF6
Ctrl
siRNA Ctrl+ Tg
siRNA BRD4 25nM + Tg
siRNA BRD4 40 nM + Tg

Figure S2

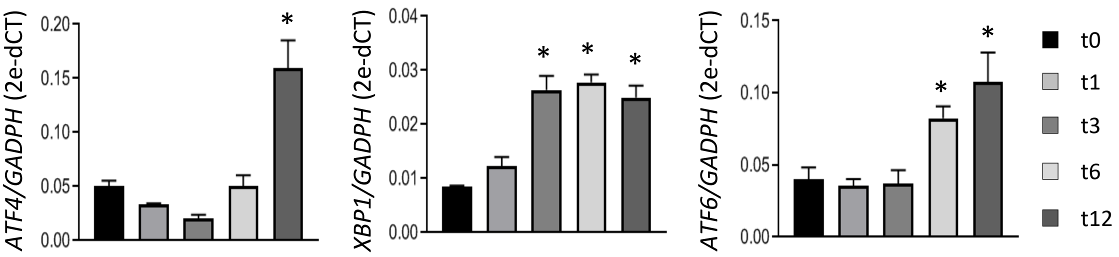
ATF4/GADPH (2e-dCT)
0.20
0.15
0.10
0.05
0.00
*
XBP1/GADPH (2e-dCT)
0.04
0.03
0.02
0.01
0.00
* * *
ATF6/GADPH (2e-dCT)
0.15
0.10
0.05
0.00
* *
t0
t1
t3
t6
t12

Figure S3

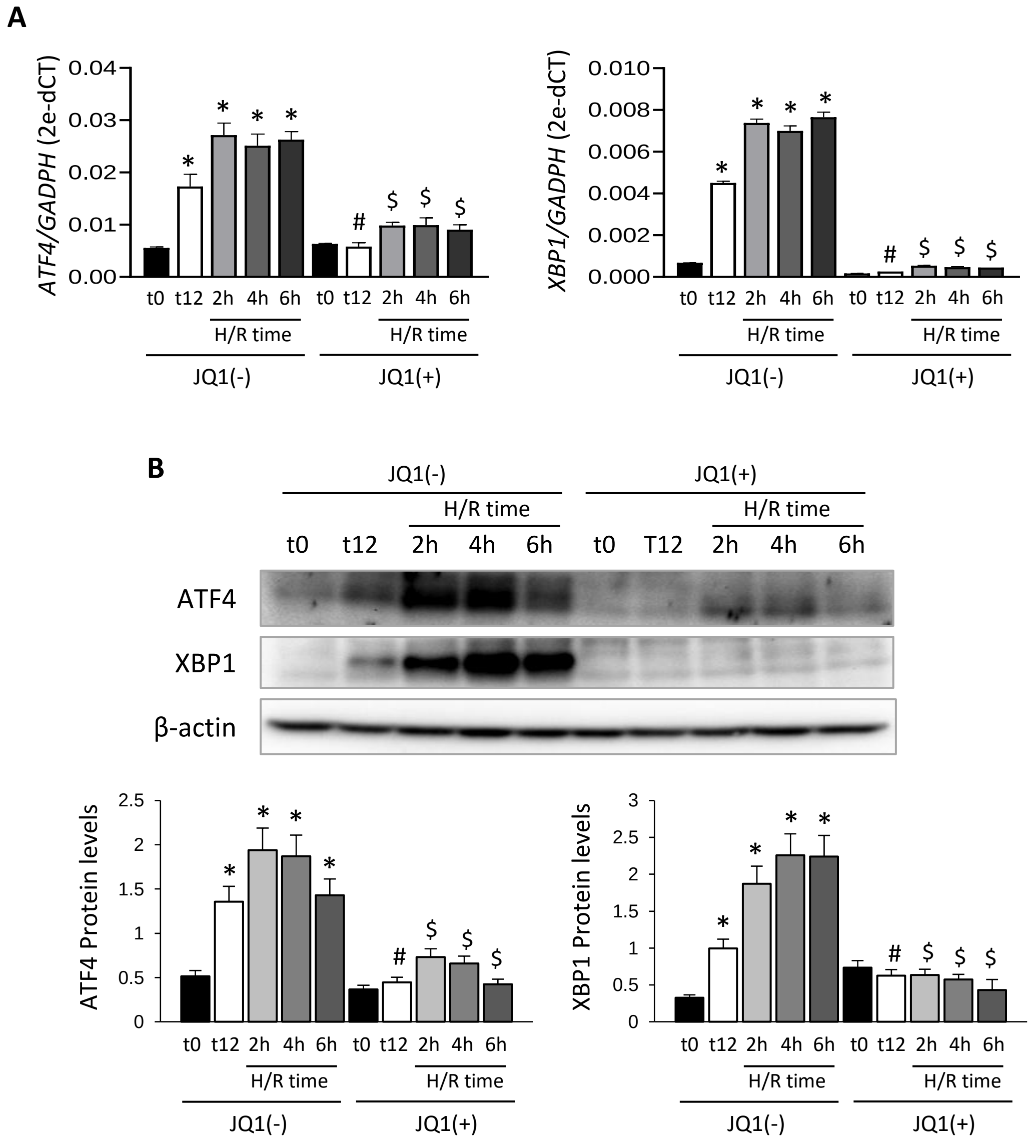

A
ATF4/GADPH (2e-dCT)
0.04
0.03
0.02
0.01
0.00
t0 t12 2h 4h 6h t0 t12 2h 4h 6h
H/R time
H/R time
JQ1(-)
JQ1(+)
XBP1/GADPH (2e-dCT)
0.010
0.008
0.006
0.004
0.002
0.000
B
JQ1(-)
JQ1(+)
H/R time
t0 t12 2h 4h 6h t0 T12 2h 4h 6h
ATF4
XBP1
β-actin
ATF4 Protein levels
2.5
2
1.5
1
0.5
0
XBP1 Protein levels
3
2.5
2
1.5
1
0.5
0

Figure S4

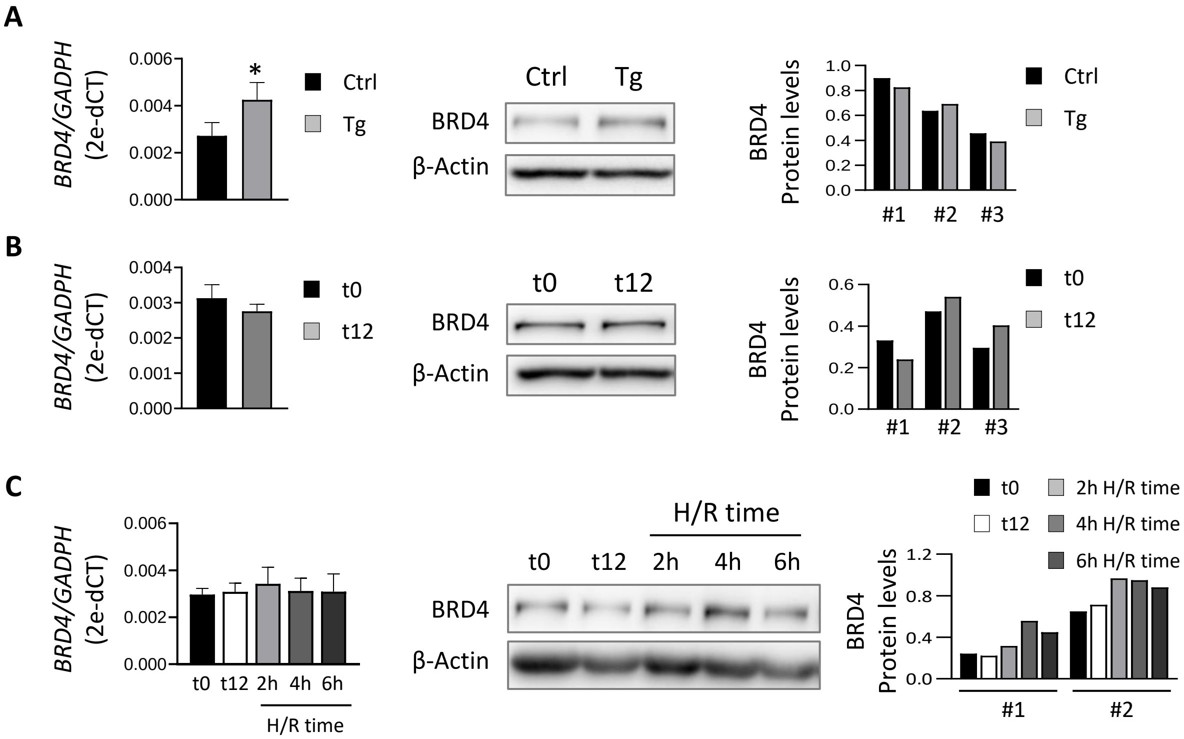
A
BRD4/GADPH (2e-dCT)
0.006
0.004
0.002
0.000
*
Ctrl
Tg
Ctrl Tg
BRD4
β-Actin
BRD4 Protein levels
1.0
0.8
0.6
0.4
0.2
0.0
#1 #2 #3
B
0.004
0.003
0.002
0.001
0.000
t0
t12
t0 t12
0.6
0.4
0.2
0.0
C
t0 t12 2h 4h 6h
H/R time
2h H/R time
4h H/R time
6h H/R time
1.2
0.8
0.4
0.0
#1 #2

Figure S5

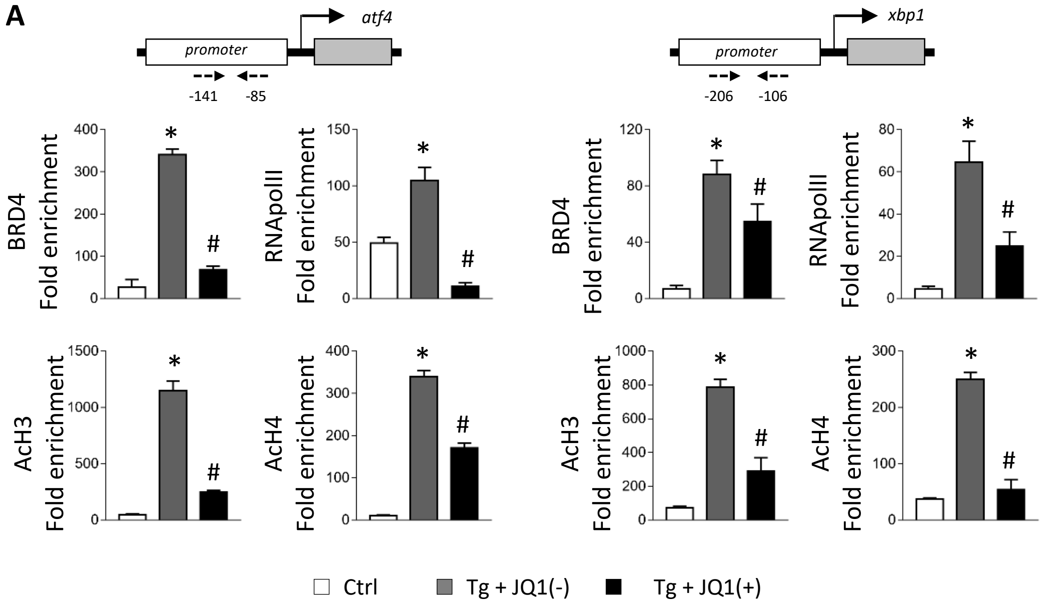
A
promoter
atf4
-141
-85
promoter
xbp1
-206
-106
BRD4 Fold enrichment
RNApolII Fold enrichment
AcH3 Fold enrichment
AcH4 Fold enrichment
Ctrl
Tg + JQ1(-)
Tg + JQ1(+)

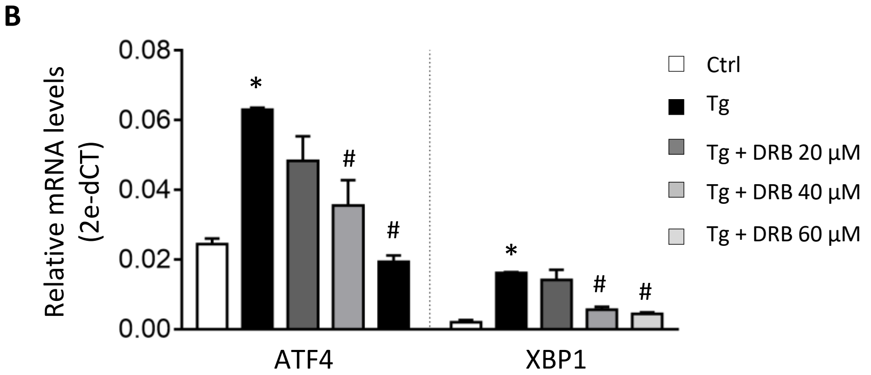
B
Relative mRNA levels (2e-dCT)
ATF4
XBP1
Ctrl
Tg
Tg + DRB 20 µM
Tg + DRB 40 µM
Tg + DRB 60 µM

Figure S6

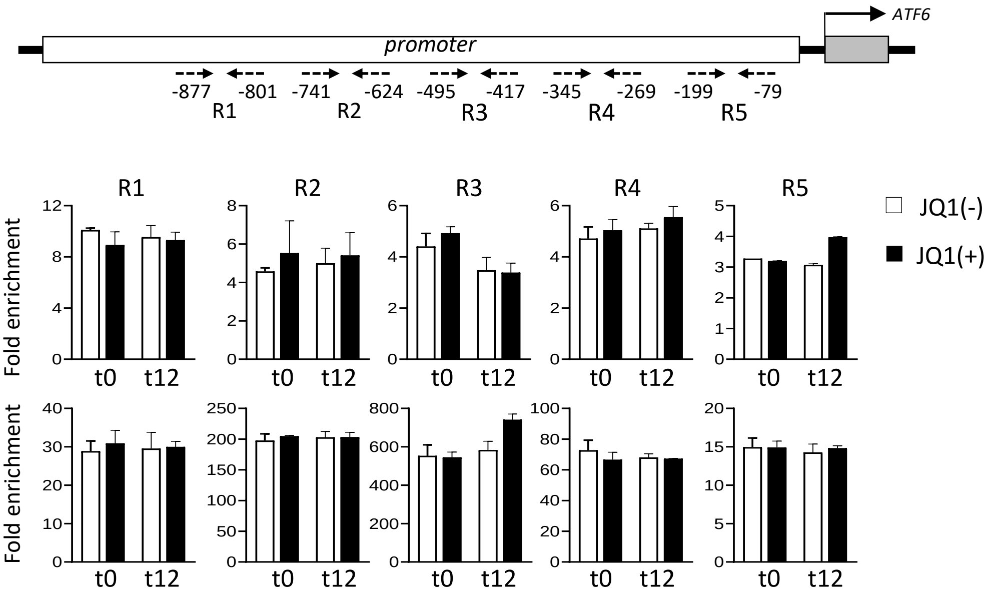

ATF6
promoter
-877
-801
-741
-624
-495
-417
-345
-269
-199
-79
R1
R2
R3
R4
R5
BRD4 Fold enrichment
Histone 3 (H3) Fold enrichment
t0
t12
JQ1(-)
JQ1(+)

Figure S7

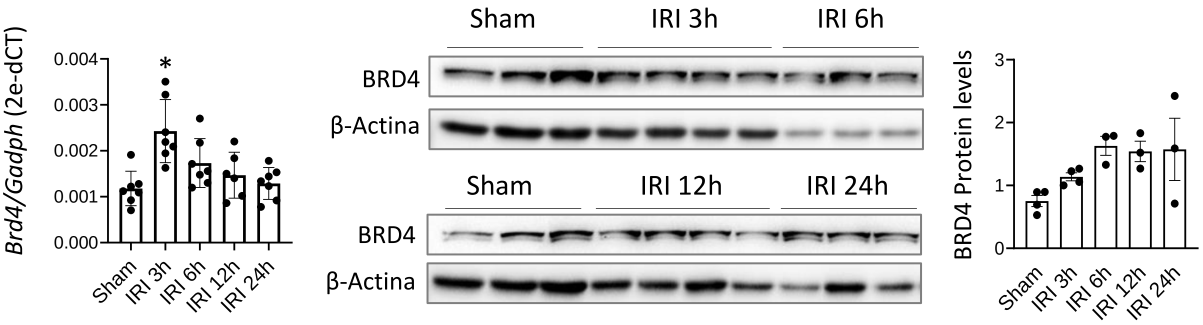

Brd4/Gadph (2e-dCT)
0.004
0.003
0.002
0.001
0.000
*
Sham
IRI 3h
IRI 6h
IRI 12h
IRI 24h
Sham
IRI 3h
IRI 6h
BRD4
β-Actina
Sham
IRI 12h
IRI 24h
BRD4
β-Actina
BRD4 Protein levels
3
2
1
0
Sham
IRI 3h
IRI 6h
IRI 12h
IRI 24h

Figure S8

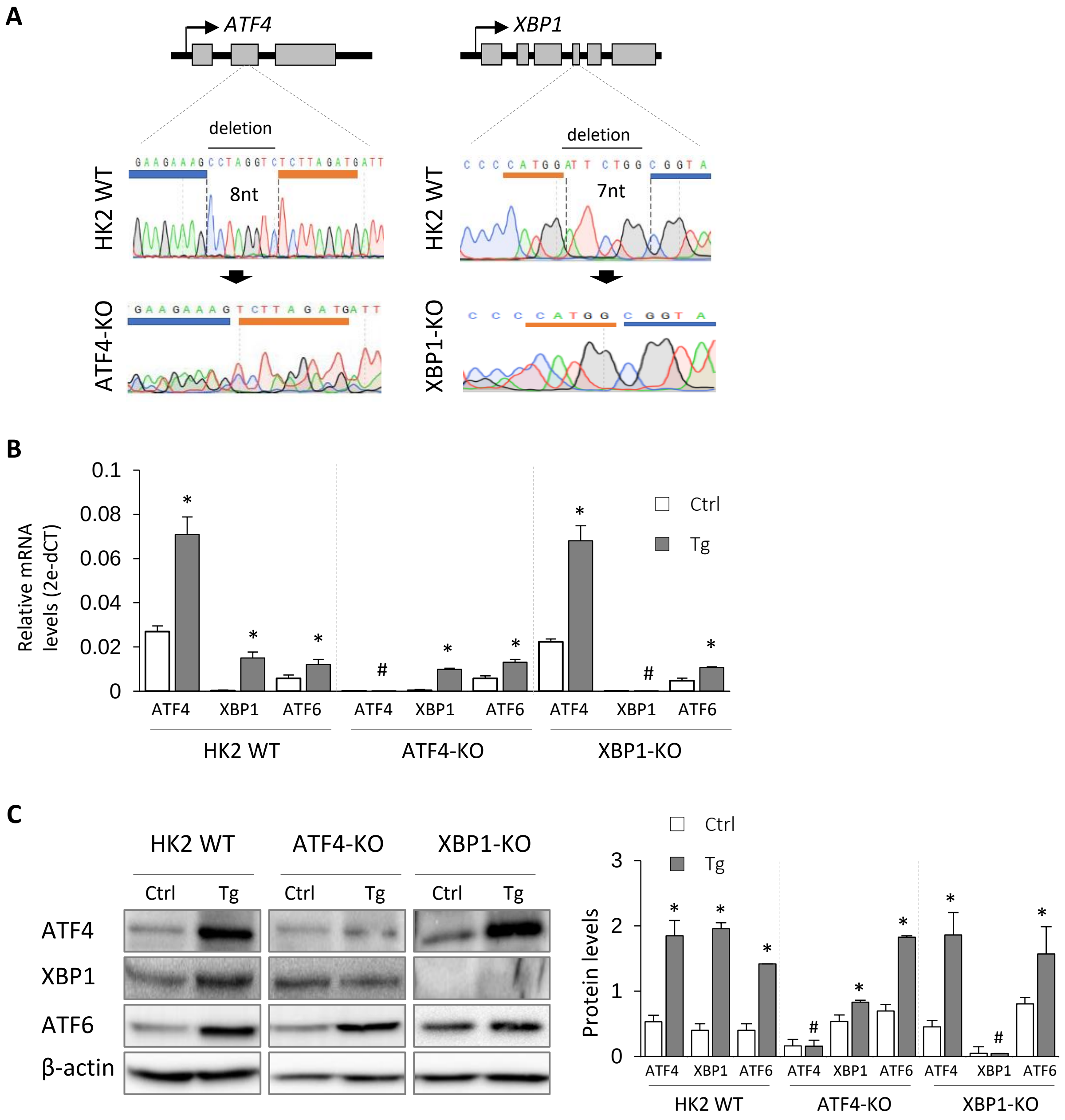

A
ATF4
XBP1
deletion
8nt
7nt
HK2 WT
ATF4-KO
XBP1-KO
B
Relative mRNA levels (2e-dCT)
0.1
0.08
0.06
0.04
0.02
0
Ctrl
Tg
ATF4
XBP1
ATF6
HK2 WT
ATF4-KO
XBP1-KO
C
HK2 WT
ATF4-KO
XBP1-KO
Ctrl
Tg
ATF4
XBP1
ATF6
β-actin
Protein levels
3
2
1
0

Figure S9

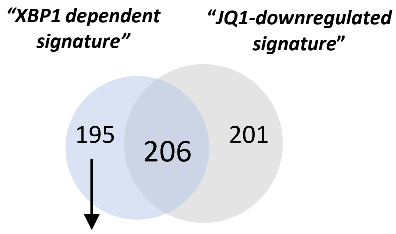

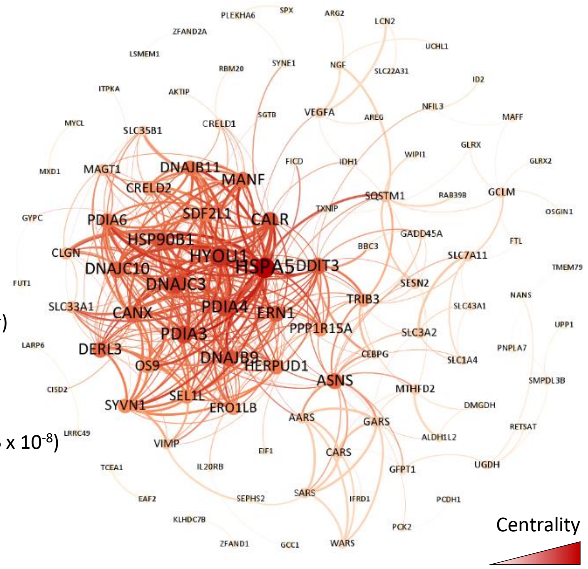

Response to ER stress (p-value = 4.08 x 10^{-17})

Ubiquitin-dependent ERAD pathway (p-value = 2.43 x 10^{-14})

Protein folding in ER (p-value = 3.13 x 10^{-13})

ER unfolded protein response (p-value = 3.88 x 10^{-9})

Protein folding (p-value = 3.92 x 10^{-8})

Retrograde protein transport, ER to cytosol (p-value = 5.55 x 10^{-8})

Response to unfolded protein (p-value = 5.08 x 10^{-5})

Regulation of autophagy (p-value = 0.0022)

Response to redox state

ER overload response

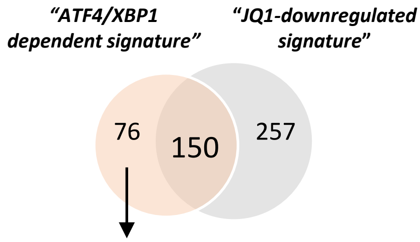

Amino acid transport (p-value = 0.0054)

L-serine biosynthetic process (p-value = 0.018)

L-lysine transmembrane transport(p-value = 0.016)

Prostaglandin biosynthetic process (p-value = 0.049)

Figure S10

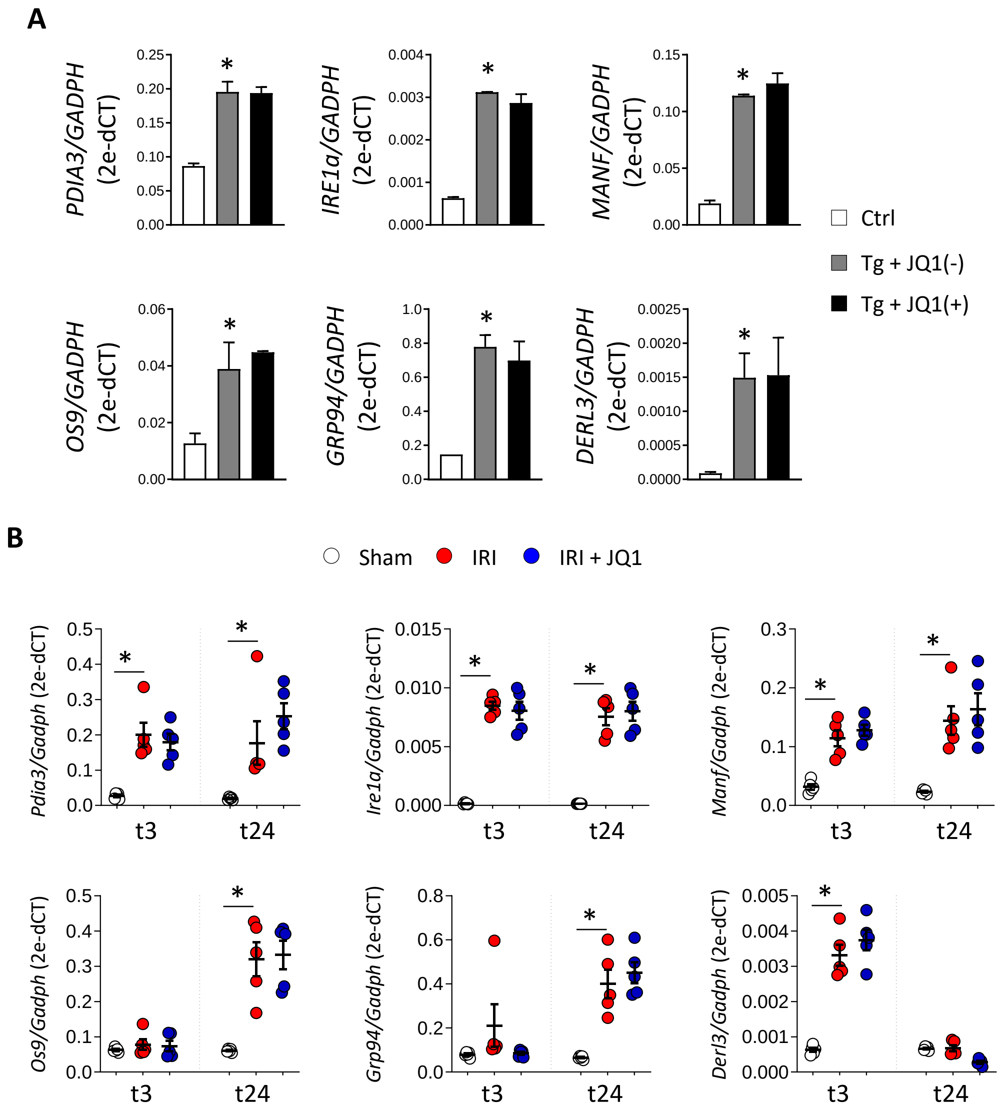

A
PDIA3/GADPH (2e-dCT)
IRE1a/GADPH (2e-dCT)
MANF/GADPH (2e-dCT)
OS9/GADPH (2e-dCT)
GRP94/GADPH (2e-dCT)
DERL3/GADPH (2e-dCT)
Ctrl
Tg + JQ1(-)
Tg + JQ1(+)
B
Sham
IRI
IRI + JQ1
Pdia3/Gadph (2e-dCT)
Ire1a/Gadph (2e-dCT)
Manf/Gadph (2e-dCT)
Os9/Gadph (2e-dCT)
Grp94/Gadph (2e-dCT)
Derl3/Gadph (2e-dCT)
t3
t24
